# Fos regulates macrophage infiltration against surrounding tissue resistance by a cortical actin-based mechanism in *Drosophila*

**DOI:** 10.1371/journal.pbio.3001494

**Published:** 2022-01-06

**Authors:** Vera Belyaeva, Stephanie Wachner, Attila Gyoergy, Shamsi Emtenani, Igor Gridchyn, Maria Akhmanova, Markus Linder, Marko Roblek, Maria Sibilia, Daria Siekhaus

**Affiliations:** 1 Institute of Science and Technology Austria, Klosterneuburg, Austria; 2 Institute of Cancer Research, Department of Medicine 1, Comprehensive Cancer Center, Medical University of Vienna, Vienna, Austria; Glasgow, UNITED KINGDOM

## Abstract

The infiltration of immune cells into tissues underlies the establishment of tissue-resident macrophages and responses to infections and tumors. Yet the mechanisms immune cells utilize to negotiate tissue barriers in living organisms are not well understood, and a role for cortical actin has not been examined. Here, we find that the tissue invasion of *Drosophila* macrophages, also known as plasmatocytes or hemocytes, utilizes enhanced cortical F-actin levels stimulated by the *Drosophila* member of the fos proto oncogene transcription factor family (Dfos, Kayak). RNA sequencing analysis and live imaging show that Dfos enhances F-actin levels around the entire macrophage surface by increasing mRNA levels of the membrane spanning molecular scaffold tetraspanin TM4SF, and the actin cross-linking filamin Cheerio, which are themselves required for invasion. Both the filamin and the tetraspanin enhance the cortical activity of Rho1 and the formin Diaphanous and thus the assembly of cortical actin, which is a critical function since expressing a dominant active form of Diaphanous can rescue the *Dfos* macrophage invasion defect. In vivo imaging shows that Dfos enhances the efficiency of the initial phases of macrophage tissue entry. Genetic evidence argues that this Dfos-induced program in macrophages counteracts the constraint produced by the tension of surrounding tissues and buffers the properties of the macrophage nucleus from affecting tissue entry. We thus identify strengthening the cortical actin cytoskeleton through Dfos as a key process allowing efficient forward movement of an immune cell into surrounding tissues.

## Abstract 2

The infiltration of immune cells into tissue underlies the establishment of tissue-resident macrophages, and responses to infections and tumors, but how do they overcome tissue barriers? This study shows that macrophages upregulate the proto-oncogene Fos, increasing the density and crosslinking of cortical actin, thereby counteracting the tension of surrounding tissues and protecting the macrophage nucleus.

## Introduction

The classical model of cell migration on a surface postulated in the 1980s by Abercrombie has been extended [1] by studies showing that migrating cells utilize diverse strategies depending on the architecture and physical properties of their three-dimensional (3D) surroundings [2]. Much of this work has been conducted in vitro, where variations in the environment can be strictly controlled. However, most 3D migration occurs within the body, and much less research has elucidated the mechanisms used to efficiently move in these diverse environments, particularly into and through tissues. Such migration is crucial for the influence of the immune system on health and disease. Vertebrate macrophages migrate into tissues during development where they take up residence, regulating organ formation and homeostasis and organizing tissue repair upon injury [3,4]. A variety of types of immune cells infiltrate into tumors and can both promote or impede cancer progression [5,6]. Responses to infection require immune cells to traverse through the vascular wall, into the lymph node, and through tissues [7]. Yet the mechanisms utilized by immune cells to allow migration into such challenging cellular environments in vivo are not well understood.

Migration in 2D and 3D environments requires actin polymerization to power forward progress. The assembly of actin at the leading edge, when coupled to Integrin adhesion to anchor points in the surrounding extracellular matrix (ECM), can allow the front of the cell to progress [8]. This anchoring also allows the contraction of cortical actin at the rear plasma membrane to bring the body of the cell forwards. But a role for cross-linked actin at the cell surface in assisting forward progress by helping to counteract the resistance of surrounding tissues and in buffering the nucleus has not been previously identified.

Our lab examines *Drosophila* macrophage migration into the embryonic germband (gb) to investigate mechanisms of immune cell tissue invasion. Macrophages, also called plasmatocytes or hemocytes, are the primary phagocytic cell in *Drosophila* and share striking similarities with vertebrate macrophages [9–13]. They are specified in the head mesoderm at embryonic stages 4 to 6 and by stage 10 start spreading along predetermined routes guided by platelet-derived growth factor-related and vascular endothelial growth factor-related factors (Pvf) 2 and 3 [9,14,15] to populate the whole embryo. One of these paths, the movement into the gb, requires macrophages to invade confined between the ectoderm and mesoderm [16,17]. The level of tension and thus apparent stiffness of the flanking ectoderm is a key parameter defining the efficiency of macrophage passage into and within the gb [16]. Penetration of macrophages into the gb utilizes Integrin, occurs normally without matrix metalloproteinases (MMPs) [17], and is even enhanced by ECM deposition [18,19] likely because the basement membrane has not yet formed at this stage [16, 20]. Thus, *Drosophila* macrophage gb invasion represents an ideal system to explore the mechanisms by which immune cells and surrounding tissues interact with one another to aid the invasion process.

Here, we sought to identify a transcription factor that could control immune cell tissue invasion and elucidate its downstream mechanisms. We identify a role for the *Drosophila* ortholog of the proto-oncogene Fos in initial entry and migration within the tissue. We find Dfos increases cortical macrophage F-actin levels through the filamin Cheerio (Cher) and a novel target, the tetraspanin TM4SF, aiding macrophages to move forward against the resistance of the surrounding tissues while buffering the nucleus.

## Results

### The transcription factor Dfos is required for macrophage germband invasion

**Fig 1:**
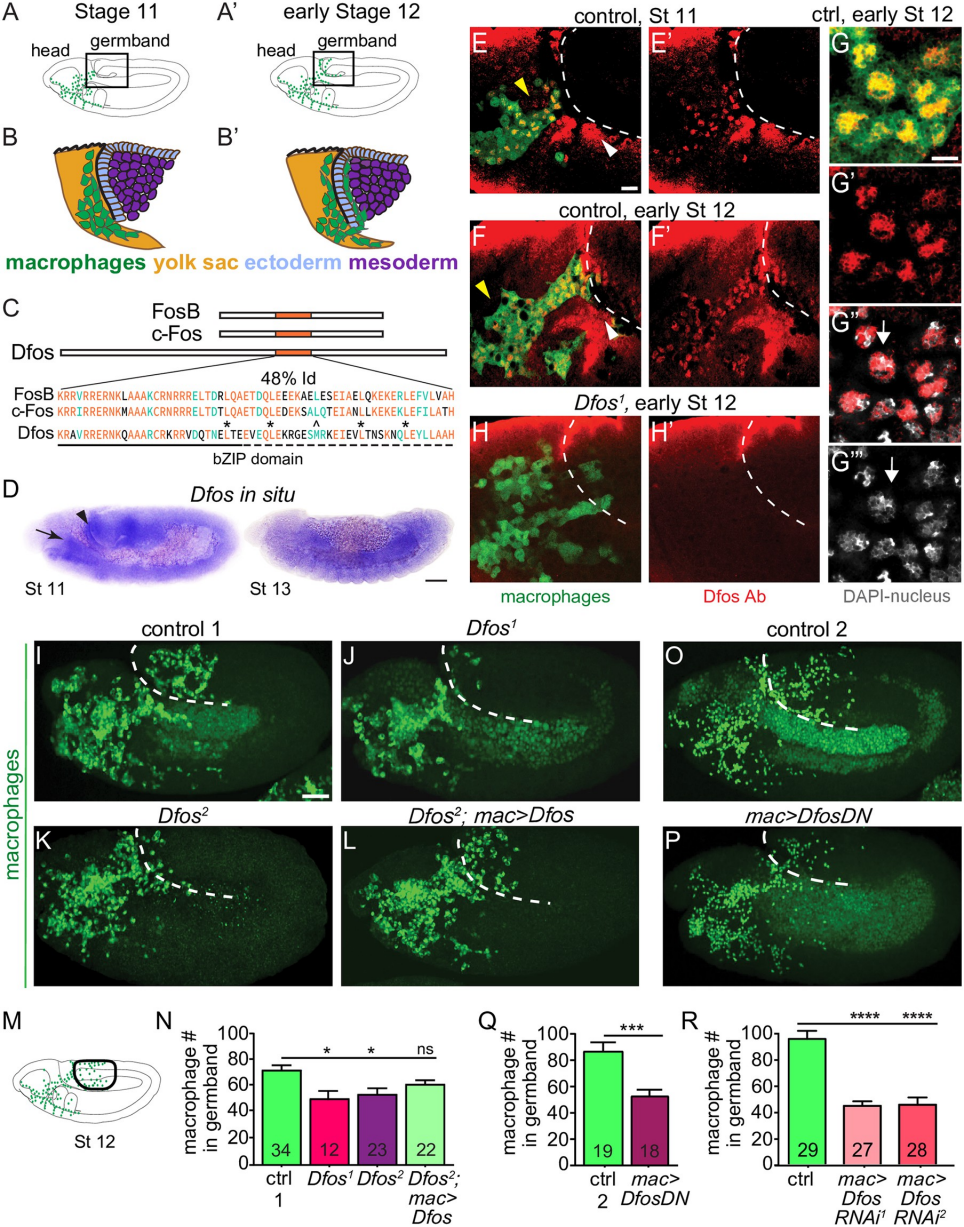
The bZIP transcription factor Dfos acts in macrophages to facilitate their migration into the gb. Schematics of lateral **(A)** stage (St) 11 and **(A’)** early St 12 embryos. The boxed region magnified below indicates where macrophages (green) invade the gb after moving there from the head **(B-B’)**. Macrophages sit on the yolk sac (yellow) next to the amnioserosa (black line) and then invade between the ectoderm (blue) and mesoderm (purple). **(C)** Dfos protein aligned with its human orthologs c-Fos and FosB; orange outlines the bZIP region that has 48% identity to both proteins: identical amino acids shown in orange, conserved ones in green. Stars indicate Leucines in the zipper; ^ the third leucine, which in Dfos is a methionine, a tolerated substitution [32]. The lower solid line indicates the basic domain and the dotted line the leucine zipper (ZIP). **(D)** In situ hybridization of St 11 and 13 embryos with a riboprobe for Dfos-RB (Fbcl0282531), which also detects all Dfos isoforms. *Dfos* RNA expression is enriched in macrophages (arrow) and the amnioserosa (arrowhead) before gb invasion but is gone thereafter. **(E-H’)** Confocal images of the boxed region in A from fixed embryos expressing *GFP* in macrophages (green) stained with a Dfos Ab (red). **(E-F’, H-H’)** A white dashed line indicates the gb edge. **(E, F)** The Dfos Ab stains (**E**) macrophages moving toward the gb at St 11 (yellow arrowheads), and **(F)** early St 12, as well as the amnioserosa (white arrowheads). **(G)** Higher magnification shows Dfos colocalizing with the nuclear marker DAPI (white). **(H)** No staining is detected in macrophages or the amnioserosa in the null *Dfos*^*1*^ mutant. **(I-L)** Lateral views of mid St 12 embryos from **(I)** the control, **(J)** the null allele *Dfos*^*1*^, **(K)** the hypomorphic allele *Dfos*^*2*^, and **(L)** *Dfos*^*2*^ with *Dfos* reexpressed in macrophages. **(M)** Schematic of St 12 embryo, gb region indicated by a black oval outline. **(N)** Quantitation reveals that both *Dfos* alleles display fewer macrophages in the gb. Reexpression of *Dfos* in macrophages in the *Dfos*^*2*^ hypomorph significantly rescues the defect. Control vs. *Dfos*^*1*^ *p* = 0.02 (30% reduction), Control vs. *Dfos*^*2*^ *p* = 0.017 (25% reduction), Control vs. *Dfos*^*2*^; *mac>Dfos p* = 0.334. **(O-P)** Lateral views of mid St 12 embryos from **(O)** the control, or **(P)** a line expressing a DN form of Dfos in macrophages. **(Q)** Quantification of macrophage numbers in the gb (see schematic) in the 2 genotypes visualized in **O**-**P**. *p* = 0.0002 (40% reduction). SD: 25, 25. **(R)** Quantification of macrophage numbers in the gb of the control and 2 different lines expressing RNAi constructs against Dfos in macrophages. Quantification of macrophage numbers in the gb for lines expressing one of 2 different *UAS-Dfos RNAi* constructs in macrophages. Control vs. *mac>Dfos RNAi*^*1*^ (TRiP HMS00254) or vs. *mac>Dfos RNAi*^*2*^ (TRiP JF02804), *p* < 0.0001 (54% or 52% reduction). SD: 32, 19, 29. The data in **Q** and **R** argue that Dfos is required within macrophages to promote gb tissue invasion. Embryos are positioned with anterior to left and dorsal up in all images, and histograms show mean + SEM throughout. Macrophages are labeled using *srpHemo-Gal4* (“mac>”) driving *UAS-GFP* in **E-H**, *UAS-GFP*::*nls* in **I-L** and *srpHemo-H2A*::*3xmCherry* in **O-R**. ***p < 0.005, **p < 0.01, *p < 0.05. One-way ANOVA with Tukey post hoc was used for **N** and **R**, and unpaired *t* test for **Q**. The embryo number analyzed is indicated within the relevant column in the graphs. Scale bar: 50 μm in **D**, 5 μm in **E-H**, and 10 μm in **I-L, O-P**. The data underlying the graphs can be found in S1 Data. bZIP, basic leucine zipper domain; DN, dominant negative; gb, germband; RNAi, RNA interference; SD, standard deviation; SEM, standard error of the mean.

To identify regulators of programs for invasion, we searched the literature for transcription factors expressed in macrophages prior to or during their invasion of gb tissues (Fig 1A–1B’). Of the 12 such factors (S1 Table, based on [21]) we focused on Dfos, a member of the Fos proto-oncogene family, assigned by the Roundup algorithm as being closest to vertebrate c-fos [22,23] (Fig 1C). Dfos contains the basic leucine zipper domain (bZIP) shown to mediate DNA binding and hetero and homo dimerization [24,25] with the third leucine replaced by a methionine, a position also altered in the *C*. *elegans* ortholog FOS-1A [26]. Embryo in situ hybridizations reveal enriched expression of the gene in macrophages at early stage 11 (Fig 1D, arrow), which is attenuated by stage 13 matching what was seen in the BDGP in situ database [27,28] https://insitu.fruitfly.org/cgi-bin/ex/report.pl?ftype=1&ftext=FBgn0001297. Antibody staining against Dfos protein appears in the nucleus in macrophages that are migrating toward the gb at stages 10 to 12 (Fig 1E–1F’ yellow arrowheads, G-G”‘ white arrows) and is still observed in stage 13 (S1A Fig). The *Dfos*^*1*^ null mutant that removes exon 1 including the translational start site [29,30] eliminates the signal in macrophages, indicating antibody specificity (Fig 1H). To determine if Dfos affects invasion, we examined the 70% of embryos that did not display developmental defects at these early stages from *Dfos*^*1*^ and the hypomorphic *Dfos*^*2*^ [30]; we quantified macrophage numbers in the gb during a defined developmental period in early stage 12 (Fig 1M). Both Dfos mutants displayed significantly reduced numbers of macrophages in the gb compared to the control (Fig 1I–1K and 1N) with normal numbers in the pre-gb zone for *Dfos*^*2*^ (S1B Fig and S1 Data). Macrophage-specific expression of *Dfos* rescues the *Dfos*^*2*^ mutant (Fig 1L and 1N). Blocking Dfos function in macrophages with a dominant negative (DN) Dfos (Fig 1O–Q) that lacks the activation domain but retains the capacity to dimerize and bind DNA [31] or 2 different RNA interferences (RNAis) against *Dfos* (Fig 1R) recapitulates the decrease in gb macrophages seen in the null while not affecting macrophage numbers in the whole embryo (S1C Fig) or along the ventral nerve cord (vnc) (S1D and S1E Fig). However, macrophages expressing DfosDN or the *Dfos* RNAis accumulate in the pre-gb area (S1F and S1G Fig), as if they are accumulating there when unable to progress further. These results argue that Dfos is required in macrophages for their migration into the gb. The tool we chose to examine this capability was DfosDN for the following reasons. Dfos and DfosDN do not appear to inhibit other bZIP proteins at higher levels of expression: Overexpressing DfosDN in the midgut does not inhibit another bZIP protein that acts there [31], and overexpressing Dfos in macrophages does not change gb numbers (S1H Fig). DfosDN should exert a quicker effect than the RNAis. And, finally, the *Dfos RNAi*s no longer exert an effect when a second UAS construct is simultaneously expressed (S1I Fig). Thus, our further experiments examining Dfos’ role in enhancing macrophage gb invasion utilized mostly the DN form.

### Dfos promotes macrophage motility and persistence during tissue entry

**Fig 2:**
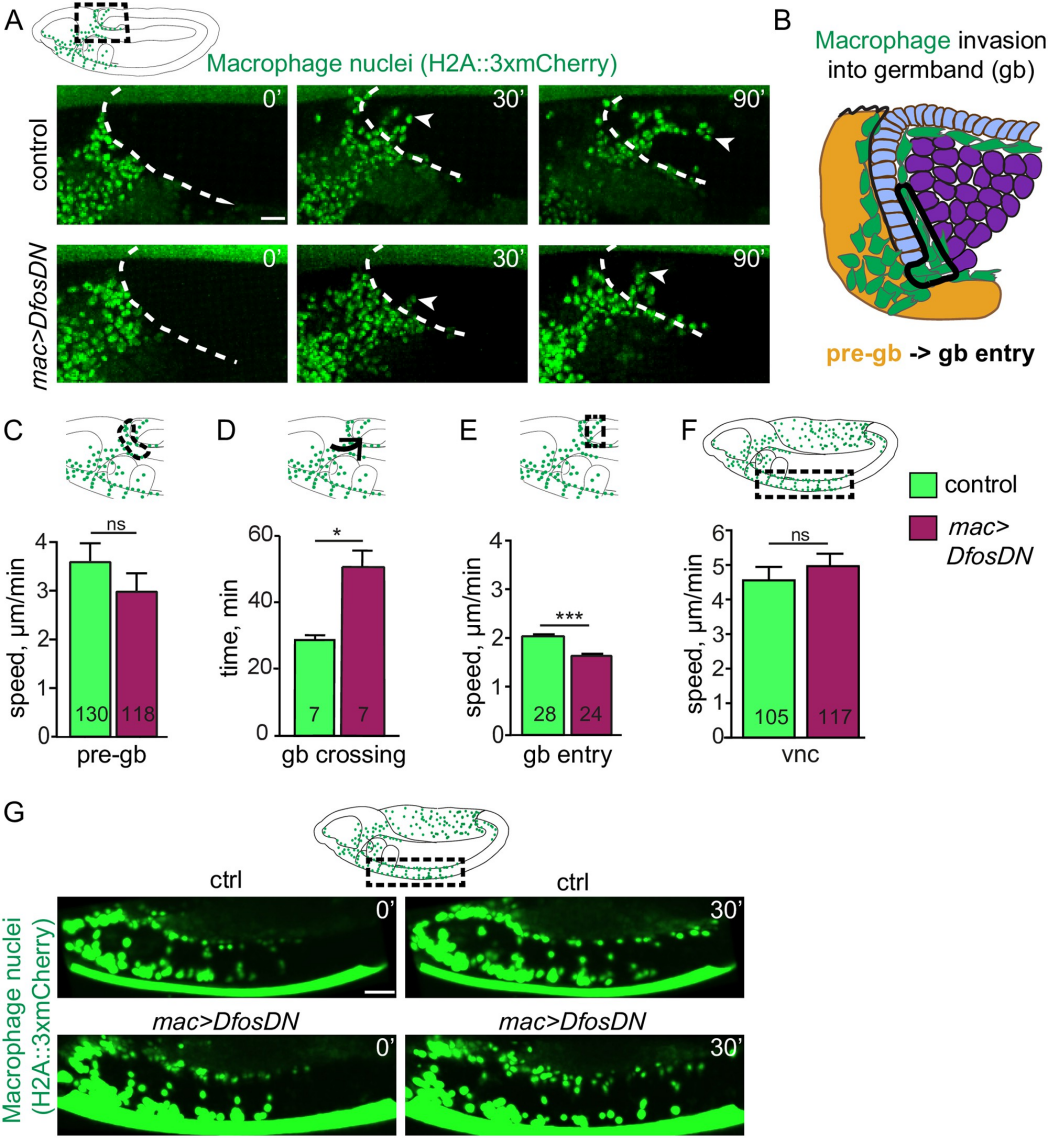
Dfos facilitates the initial invasion of macrophages into the gb tissue. (**A**) Movie stills of control embryos and those expressing DfosDN in macrophages (green, nuclei labeled using *srpHemo-H2A*::*3xmCherry*). Area imaged corresponds to the black dashed square in the schematic above. The gb border is outlined with a white dashed line. The first entering macrophage is indicated with a white arrowhead, and time in minutes in the upper right corner. (**B**) Detailed schematic showing the different zones for which the parameters of macrophage gb invasion were quantified. The pre-gb area is shown in yellow, the gb entry zone is outlined in a solid line. **(C)** Macrophage speed in the pre-gb area was not significantly changed in macrophages expressing DfosDN (3.00 μm/min) compared to the control (3.61 μm/min), *p* = 0.58. (**D**) Quantification shows a 68% increase in the total gb crossing time of DfosDN expressing macrophages compared to the control. Total gb crossing time runs from when macrophages have migrated onto the outer edge of the gb ectoderm, aligning in a half arc, until the first macrophage has translocated its nucleus into the gb ecto–meso interface. *p* = 0.008. SD: 4, 14. (**E**) DfosDN expressing macrophages displayed a significantly reduced speed (1.53 μm/min) at the gb entry zone compared to the control (1.98 μm/min), *p* = 1.11e^−06^. SD: 2, 2. (**F**) Macrophages expressing DfosDN in a Stage 13 embryo move with unaltered speed along the vnc in the region outlined by the dashed black box in the schematic above (4.93 μm/min), compared to the control (4.55 μm/min), *p* = 0.64. Corresponding stills shown in **(G**) Macrophages are labeled by *srpHemo-Gal4* driving *UAS-GFP*::*nls*. ***p < 0.005, **p < 0.01, *p < 0.05. Unpaired *t* test used for **C-F**, a Kolmogorov–Smirnov test for **D**. For each genotype, the number of tracks analyzed in **C** and **F** and the number of macrophages in **D-E** are indicated within the graph columns. Tracks were obtained from movies of 7 control and 7 *mac>DfosDN* expressing embryos in panel **D**, 3 each in **C, F**, and 4 each in **E**. Scale bar: 10 μm. The data underlying the graphs can be found in S1 Data. gb, germband; ns, not significant; SD, standard deviation; vnc, ventral nerve cord.

To examine the dynamic effects of Dfos on tissue invasion, we performed live imaging and tracking of macrophages. We visualized macrophage nuclei with *srpHemo-H2A*::*3xmCherry* [33] in either a wild-type or *mac>DfosDN* background, capturing the initial stage of invasion (Fig 2A and S1 Movie). The speed of macrophages moving in the area neighboring the gb prior to invasion was not significantly changed (pre-gb, Fig 2B and 2C). However, the first *mac>DfosDN* macrophage to enter is delayed by 20 minutes in crossing into the gb (Fig 2D). *mac>DfosDN* macrophages also displayed reduced speed and directional persistence during entering as well as while moving along the first 20 μm of the ectoderm–mesoderm interface (gb entry, Fig 2E and S2A Fig). Macrophages in the *Dfos*^*2*^ mutant largely mirrored this phenotype but displayed slower movement in the pre-gb zone (S2B and S2C Fig and S2 Movie) neighboring the amnioserosa in which Dfos is also expressed (Fig 1D, black arrowhead, Fig 1E and 1F, white arrowheads), likely causing a nonautonomous effect. Macrophages expressing DfosDN moved with unaltered average speed as they spread out along the noninvasive route of the vnc (Fig 2F and 2G and S3 Movie), albeit with reduced directional persistence (S2A Fig). We thus conclude from live imaging that Dfos in macrophages aids their initial invasive migration into the gb, increases their speed within the gb, and does not underlie their progress along the vnc.

### Dfos modulates Filamin and Tetraspanin to aid gb tissue invasion

**Fig 3:**
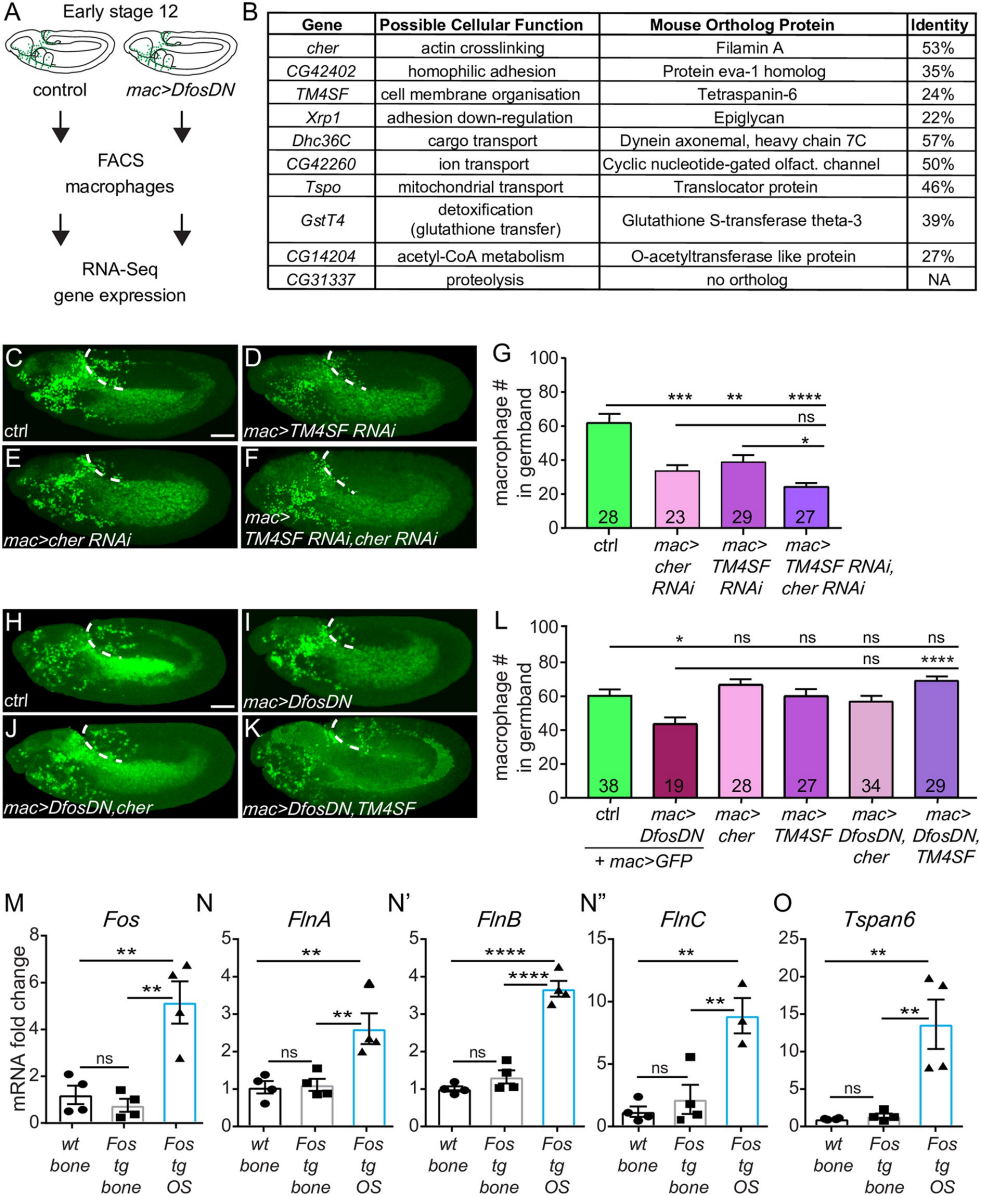
Dfos regulates macrophage gb invasion through cytoskeletal regulators: The filamin Cher and the tetraspanin TM4SF. **(A)** Schematic representing the pipeline for analyzing mRNA levels in FACS-sorted macrophages. **(B)** Table of genes down-regulated in macrophages expressing DfosDN. Genes are ordered according to the normalized *p*-value from the RNA sequencing. The closest mouse protein orthologs were found using UniProt BLAST; the hit with the top score is shown in the table. **(C-F)** Lateral views of representative St 12 embryos in which the 2 targets with links to actin organization, (**D**) the tetraspanin TM4SF and (**E**) the filamin Cher, have been knocked down individually or (**F**) together, along with the control (**C**). Scale bar: 50 μm. **(G)** Quantification shows that the number of macrophages in the gb is reduced in embryos expressing RNAi against either *cher (KK 107451*) or *TM4SF* (*KK 102206*) in macrophages, and even more strongly affected in the double RNAi of both. Control vs. *cher RNAi p* = 0.0005 (46% reduction). Control vs. *TM4SF RNAi p* = 0.009 (37% reduction), Control vs. *cher/TM4SF RNAi p* > 0.0001 (61% reduction). *cher RNAi* vs. *TM4SF RNAi p* = 0.15. SD: 29, 23, 17, 12. **(H-K)** Lateral views of a representative St 12 embryo from (**H**) the control, as well as embryos expressing DfosDN in macrophages along with either (**I**) GFP, (**J**) Cher, or (**K**) TM4SF. (**L**) Quantification shows that overexpression of TM4SF in DfosDN expressing macrophages restores their normal numbers in the gb. Overexpression of Cher in this background shows a strong trend toward rescue but did not reach statistical significance. Control vs. *DfosDN p* = 0.015 (28% reduction); Control vs. *cher p* = 0.74; Control vs. *TM4SF p* > 0.99; *DfosDN* vs. *DfosDN cher p* = 0.14; DfosDN vs. *DfosDN*, *TM4SF p* < 0.0001; Control vs. *DfosDN cher p* = 0.97; Control vs. *DfosDN TM4SF p* = 0.35. SD: 22, 16, 16, 21, 22, 13. **(M-O)** q-PCR analysis of mRNA extracted from the bones of mice that are wt, tg for *Fos* controlled by a Major Histocompatibility promoter and viral 3′ UTR elements, and those in which such c-Fos transgenesis has led to an OS. Analysis of mRNA expression shows that higher levels of **(M)** *Fos* correlate with higher levels of **(N-N”)** FlnA-C, and **(O)** Tspan6 in OS. *p*-values = 0.86, 0.001, 0.003, SD: 0.7, 0.6, 0.3 in **M**, 0.98, 0.009, 0.007 and 0.4, 0.2, 1.5 in **N**, 0.39, <0.0001, <0.0001 and 0.2, 0,3, 1.1 in N’, 0.76, 0.005, 0.002 and 0.8, 2.3, 2.4 in N”, 0.99, 0.004, 0.003 and 0.1, 0.2, 0.2 in O. Scale bar: 50 μm. Macrophages are labeled using either **(C-F)** *srpHemo-H2A*::*3xmCherry* or **(H-K)** *srpHemo-Gal4* (“*mac>*”) driving *UAS-mCherry*::*nls*. ***p < 0.005, **p < 0.01, *p < 0.05. Unpaired *t* test or one-way ANOVA with Tukey post hoc were used for statistics. Each column contains the number of analyzed embryos. The data underlying the graphs can be found in S1 Data. Cher, Cheerio; gb, germband; ns, not significant; RNAi, RNA interference; OS, osteosarcoma; tg, transgenic; wt, wild type.

To identify Dfos targets that promote macrophage invasion, we FACS isolated macrophages from wild-type and *mac>DfosDN* embryos during the time when invasion has just begun and conducted RNA sequencing of the corresponding transcriptomes (Fig 3A and S2 Data). We first assessed reads that map to Dfos, which can correspond to both endogenous and DfosDN mRNA; we found a 1.6-fold increase in the presence of the one copy of DfosDN in this line, arguing that this transgene is expressed at levels similar to each endogenous copy of Dfos and is unlikely to produce extraneous effects. We then examined genes that displayed a log_2_ fold change of at least 1.5 with an adjusted *p*-value less than 0.05 in the presence of DfosDN. Ten genes were down-regulated (Fig 3B and S3A and S3B Fig) and 9 up-regulated by DfosDN (S2 Table). Up-regulated genes in DfosDN encoded mostly stress response proteins, consistent with the role previously demonstrated for fos in *C*. *elegans* in suppressing stress responses [34]. We concentrated on the down-regulated class. Of these, we focused on the actin cross-linking filamin Cher and the tetraspanin TM4SF from a group that can form membrane microdomains that affect signaling and migration [35,36]. No known role for TM4SF had been previously identified in *Drosophila*. To determine if these Dfos targets were themselves required for invasion, we knocked down Cher and TM4SF through RNAi individually or simultaneously and observed significantly reduced macrophage numbers in the gb, particularly upon the knockdown of both targets simultaneously (Fig 3C–3G) while not affecting macrophage numbers in the pre-gb zone (S3D Fig) or on the vnc (S3E Fig). Overexpression of Cher or TM4SF along with *DfosDN* in macrophages increased the mean macrophage numbers in the gb, and overexpression of TM4SF rescued the *DfosDN* macrophage invasion defect (Fig 3H–3L). Expression of a GFP control did not restore macrophage invasion indicating that the rescue we observed through Cher or TM4SF expression was not due to promoter competition leading to reductions in DfosDN expression. We conclude that Dfos aids macrophage gb invasion by increasing the mRNA levels of the filamin actin cross-linker Cher and the tetraspanin TM4SF.

### In murine osteosarcoma, c-fos mRNA level increases correlate with those of Filamins and Tetraspanin-6

To determine if these Dfos targets in *Drosophila* could also be Fos targets in vertebrate cells, we utilized a well-established murine transgenic model that overexpresses c-fos. In these mice, transgenic c-fos expression from viral 3′ UTR elements in osteoblasts (the bone forming cells) leads to osteosarcoma (OS) development accompanied by a 5-fold increase in c-fos mRNA expression (Fig 3M) [37]. We examined by qPCR the mRNA levels of our identified Dfos targets’ orthologs, comparing their levels in OS (Fos tg OS) to neighboring, osteoblast-containing healthy bones from Fos tg mice (Fos tg bone) and control bones from wild-type mice (wt bone). We saw 2.5- to 8-fold higher mRNA levels of the 3 murine Filamin orthologs (Fig 3N–3N”) and a 15-fold increase in Tetraspanin-6 (Fig 3O) in OS cells. mRNA levels of several of the orthologs of other Dfos targets we had identified showed less strong inductions or even decreases; the Glutathione S transferase Gstt3 and the Slit receptor Eva1c increased 4- and 2.8-fold, respectively, while the mitochondrial translocator Tspo was 25% lower (S3F–S3I Fig). These results suggest that Dfos’s ability to increase mRNA levels of 2 key functional targets for migration, a Filamin and a Tetraspanin, is maintained by at least one vertebrate fos family member.

### Dfos increases assembly of cortical actin through Cheerio and TM4SF to aid macrophage invasion

**Fig 4:**
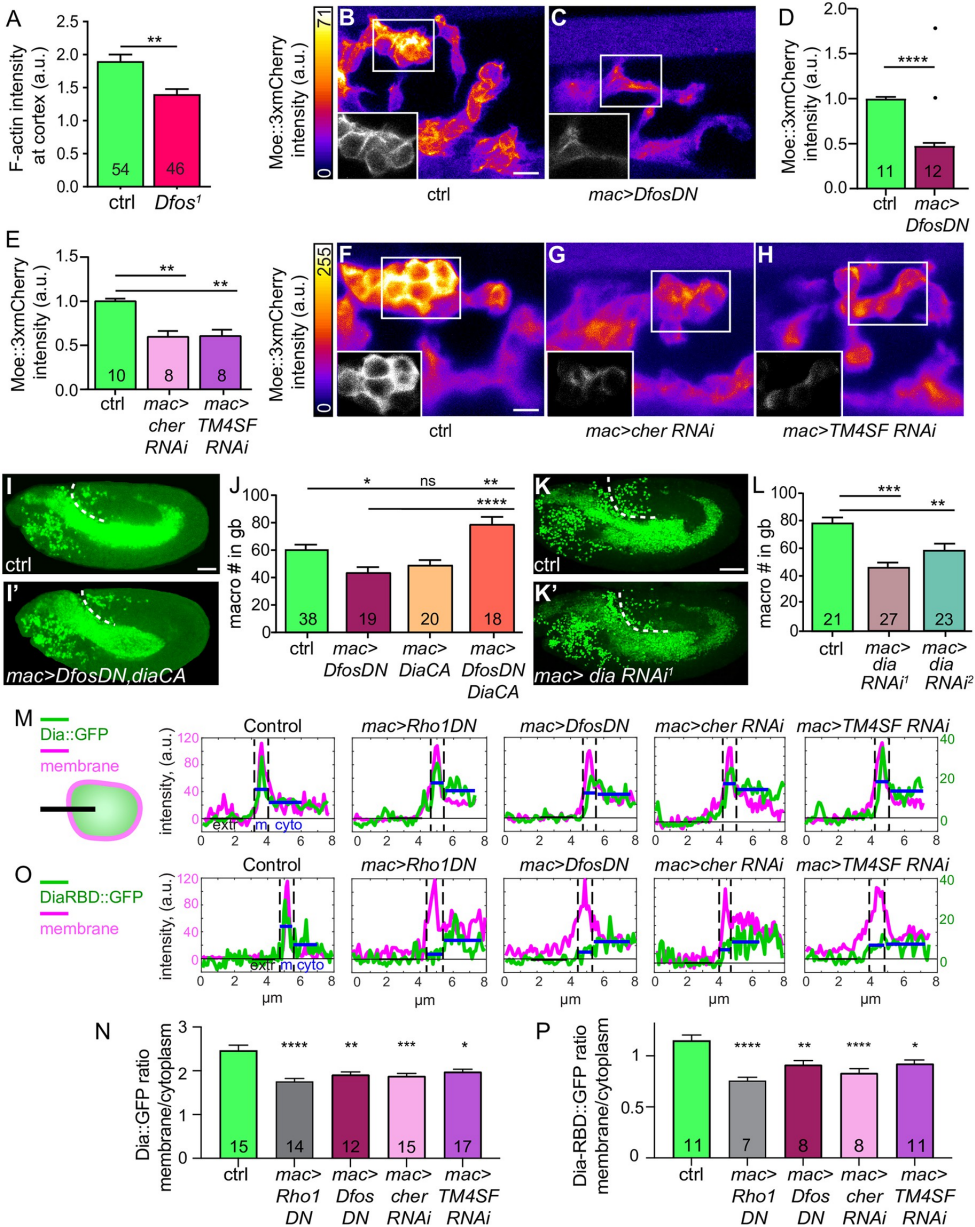
Dfos increases Rho1-GTP, the formin Diaphanous and actin at the cortex through Cher and TM4SF. **(A)** Quantification of phalloidin intensity to detect F actin at the macrophage–macrophage contacts in Stage 11/12 *Dfos*^*1*^ embryos. F-actin is strongly reduced at these homotypic contacts. **(B-C)** Representative confocal images of live embryos expressing in invading macrophages the F-actin binding and homodimerizing portion of Moesin (*srpHemo-moe*::*3xmCherry)* to label F-actin, presented as a maximum z-projection. Relative Moe-3xmCherry intensity is indicated with a pseudo-color heat map as indicated on the left, with yellow as the highest levels and dark blue as the lowest as indicated in the calibration bar to the left. Insets in the bottom left corner of each panel show a grayscale single z-plane corresponding to the white box in the main image. Embryo genotype indicated below. Strong reductions in cortical actin are observed in macrophages expressing DfosDN compared to the control. **(D-E)** Quantification of the macrophage Moe:3xmCherry intensity as a measure of cortical F-actin, normalized to the average fluorescence intensity of the control per batch. **(D)** Quantification shows that macrophages expressing DfosDN display a 53% reduction in Moe::3xmCherry intensity compared to the control when the 2 outliers shown as single dots are excluded, 37% if they are included. Outliers identified by 10% ROUT. n of ROIs analyzed = 650 for control, 687 for *DfosDN*. *p* = 0.0007 for analysis including outliers (Kolmogorov–Smirnov) and *p* < 0.0001 for analysis excluding outliers (Welch’s *t* test). SD: 0.2, 0.4. **(E)** Quantification reveals that macrophage expression of an RNAi against either *cher* or *TM4SF*, the 2 genes whose expression is reduced in *DfosDN*, also results in a decrease of Moe::3xmCherry intensity (by 40% each). n of ROIs analyzed = 549 for control, 423 for *cher RNAi*, 306 for *TM4SF RNAi*. Control vs. *cher RNAi p* = 0.006. Control vs. *TM4SF p* = 0.003. SD: 0.2, 0.3, 0.2. **(F-H)** Images and representation as in B-C. Strong reductions in cortical actin are observed in macrophages expressing *cher RNAi* or *TM4SF RNAi* compared to the control. **(I-I’)** Representative confocal images of St 12 embryos from the control and a line in which macrophages express DfosDN and a CA form of the formin Dia to restore cortical actin polymerization. **(J)** Quantification shows that while macrophage expression of DiaCA does not significantly affect the number of macrophages in the gb, expressing it in a DfosDN background rescues macrophage gb invasion. Control vs. *DfosDN p* = 0.017 (28% reduction), Control vs. *diaCA p* = 0.18, Control vs. *DfosDN*, *diaCA p* = 0.010, *DfosDN* vs. *DfosDN*, *diaCA p* < 0.0001. SD: 22, 16, 16, 24. (**K–K’**) Representative confocal images of St 12 embryos from the control and from a line expressing an RNAi against *dia* in macrophages. **(L)** Quantification of 2 RNAi lines against *dia* expressed in macrophages shows a 37% and 21% reduction in macrophage numbers in the gb compared to control. Control vs. *dia RNAi*^*1*^ (TRiP HMS05027) *p* < 0.0001; control vs. *dia RNAi*^*2*^ (TRiP HMS00308) *p* = 0.0008. SD: 13, 20, 22. (**M, O**) Examples of line profiles used for the determination of the membrane-to-cytoplasmic ratio of Dia in panel N and the Rho1 activity sensor DiaRBD in panel P. Line intensity profiles from fixed Stage 11 embryos of (M) Dia::GFP or (O) DiaRBD::GFP (green) and membrane Myr::Tomato (magenta) across the outward facing edge of groups of macrophages sitting within approximately 40 μm of the gb that expressed either lacZ (Control), Rho1DN, DfosDN, *cher RNAi*, or *TM4SF RNAi* as shown in the schematic in M. Line length approximately 8 μm. Blue lines indicate mean GFP intensity on the membrane and in cytoplasm. (**N, P**) Quantification of membrane-to-cytoplasmic intensity ratio of (N) Dia::GFP or (P) the Rho1 activity sensor DiaRBD::GFP expressed in macrophages under UAS control along with either lacZ (control, *n* = 233 from 15 or *n* = 158 line scans from 11 embryos), Rho1DN (*n* = 212 from 14 or *n* = 123 from 7), DfosDN (*n* = 237 from 12 or *n* = 135 from 8), *cher RNAi* (*n* = 252 from 13 or *n* = 128 from 8), *TM4SF RNAi* (*n* = 279 from 17 or *n* = 205 from 11). Control vs. Rho1DN ****p < 0.0001 (29% (N), 34% (P) reduction), Control vs. DfosDN **p = 0.0037 (23% (N), 21% (P) reduction), Control vs. *cher RNAi* ***p = 0.0007, 24% reduction (N) or ****p < 0.0001, 28% reduction (P), Control vs. *TM4SF RNAi* *p = 0.024 or 0.026 (20% reduction). SD: 1.9, 0.9, 1.0, 0.9, 1.0 in N; 0.7, 0.5, 0.5, 0.5, 0.4 in P. Macrophages are labeled using either *srpHemo-Gal4* driving *UAS-mCherry*::*nls* **(I-I’)**, *srpHemo-H2A*::*3xmCherry* **(K-K’)**. *srpHemo-moe*::*3xmCherry*, *srpHemo-Gal4* (*mac>)* crossed to **(B)** *UAS-GFP* as a Control, **(C)** *UAS-DfosDN*, **(F)** *w*^−^ Control, **(G)** *UAS-cher RNAi* (KK 107451), **(H)** *UAS-TM4SF RNAi* (KK 102206). *srpHemo-GAL4 UAS-Myr*::*tdTomato UAS-dia*::*GFP* (M, O) *or UAS-diaRBD*::*GFP* (N, P) crossed to *UAS-lacZ* as a Ctrl, *UAS-Rho1DN* or the lines indicated above. ****p < 0.0001, ***p < 0.005, **p < 0.01, *p < 0.05. Unpaired *t* test used for **A**. Welch’s *t* test of normalized average mean intensity per embryo for **D** with the 2 indicated outliers excluded, for statistical assessment. One-way ANOVA with Tukey post hoc for **E**, **J, L**. Kruskal–Wallis for **N, P**. The number of analyzed **(A)** macrophage–macrophage junctions, or **(D-E, J, L, N, P)** embryos is shown in each column. Scale bar 10 μm in **(B-C, F-H)**, 50 μm in (**I, K**). The data underlying the graphs can be found in S1 Data. CA, constitutively active; Cher, Cheerio; ctrl, control; gb, germband; ns, not significant; RNAi, RNA interference; ROI, region of interest.

We wished to determine what cellular properties Dfos could affect through such targets to facilitate *Drosophila* macrophage invasion. Given Cher’s known role as an actin cross-linker, we stained embryos with phalloidin to detect F-actin. Line scan analysis revealed reduced intensity at the macrophage cortex in fixed *Dfos*^*1*^ mutant embryos in the pre-gb and gb entry zone (Fig 4A). To examine this in invading *mac>DfosDN* macrophages within live embryos, we utilized a *srpHemo-moe*::*3xmCherry* reporter, which marks cortical F-actin [38,39] only in macrophages and observed a reduction of 53% (Fig 4B–4D) in its signal. We saw no change by western analysis in the levels of the Moe::3xmCherry protein itself upon DfosDN expression (S4A and S4A’ Fig). We hypothesized that the changes in macrophage cortical actin we observed in the *mac>DfosDN* could be due to the lower levels of Cher and/or TM4SF mRNA. Indeed, we observed reductions in Moe::3xmCherry all around the edge of invading macrophages in live embryos expressing RNAi against Cher or TM4SF in macrophages (Fig 4E–4H). To test if a decrease in actin assembly could underlie the reduced tissue invasion of *mac>DfosDN* macrophages, we forced cortical actin polymerization by expressing a constitutively active version of the formin Diaphanous (DiaCA), in which Dia’s inhibitory autoregulatory domain has been deleted, allowing active Dia to localize to the macrophage cortex [40]. Indeed, expressing DiaCA in macrophages completely rescued the *Dfos*^*1*^, *Dfos*^*2*^ (S4B Fig), and *mac>DfosDN* invasion defect (Fig 4I and 4J). Given that Dia, like Dfos, does not affect general macrophage migratory capacities along the vnc [41], we examined if Dia might normally play a role in invasion. We utilized 2 RNAis against Dia and observed decreased macrophage numbers in the gb in each (Fig 4K and 4L) with no effect on numbers in the pre-gb (S4C Fig) or on the vnc (S4D Fig). These results argue that Dfos aids invasion by increasing levels of TM4SF and Cher to enhance assembly of actin around the surface of the macrophage.

### Dfos stimulates the cortical activity of Rho1 and Diaphanous through its targets TM4SF and Cheerio

We hypothesized that Dfos and its targets enhance cortical actin assembly by affecting Dia. We had observed no change in Dia’s mRNA levels (S3C Fig) upon DfosDN expression and thus examined localization of Dia protein. We expressed Dia::GFP [42] in macrophages along with myristoylated Tomato (Myr::Tomato) to mark the membrane and quantified intensity profiles of linescans across the membrane in various genetic backgrounds, assessing the ratio of membrane/cytoplasmic Dia (Fig 4M and S4E Fig). Dia’s autoinhibition negatively regulates its cortical localization and activity in *Drosophila* macrophages [40,43]. For mDia, binding to activated Rho GTPases as well as to other unknown membrane associated proteins can release this autoinhibition [44]. *Drosophila* Rho1 has been shown to directly bind Dia lacking its autoinhibitory domain [45]. As predicted by these prior results, upon the expression of Rho1DN, we observed a significant reduction, by 29%, in the enrichment of Dia at the cortex compared to the control (mem/cyto = 2.46 in control, 1.76 for Rho1DN) (Fig 4N). We found that expressing either DfosDN or RNAis against Cher or TM4SF resulted in a significant reduction of cortical Dia, 80%, 83%, and 70%, respectively, as strong as that seen upon Rho1DN expression (mem/cyto = 1.9, 1.88, 1.97). To assess if this effect of the Dfos pathway on Dia could be due to an effect on Rho activity itself, we expressed a sensor of active Rho1, the Rho1 binding domain of Dia (DiaRBD::GFP) [46], in macrophages along with Myr::Tomato to delineate the plasma membrane and quantified intensity profiles of linescans across the membrane in various genetic backgrounds as above (Fig 4O and S4F Fig). To validate the assay, we expressed Rho1DN and found, as expected, a significant reduction, by 34%, in the enrichment of the Rho1 sensor DiaRBD at the cortex compared to the control (mem/cyto = 1.15 in control, 0.76 for Rho1DN) (Fig 4P). Expressing either DfosDN or RNAis against the filamin Cher or the tetraspanin TM4SF also resulted in a significant reduction of cortical DiaRBD, by 62%, 82%, and 59%, respectively, as much as that seen upon Rho1DN expression (mem/cyto = 0.91, 0.83, 0.92, respectively). The lower Rho1 activity we observed in the absence of the Dfos pathway could be a result of reduced Rho1 GEF recruitment, as Filamin has been shown to bind the Rho GTPase GEFs Trio and Vav2 [47,48] and a tetraspanin can recruit a filamin [49,50]. Our data argue that higher levels of the Dfos targets TM4SF and Cher increase Dia localization at the cortex and thus stimulate cortical actin assembly, at least partially through increased Rho1 activity.

**Fig 5:**
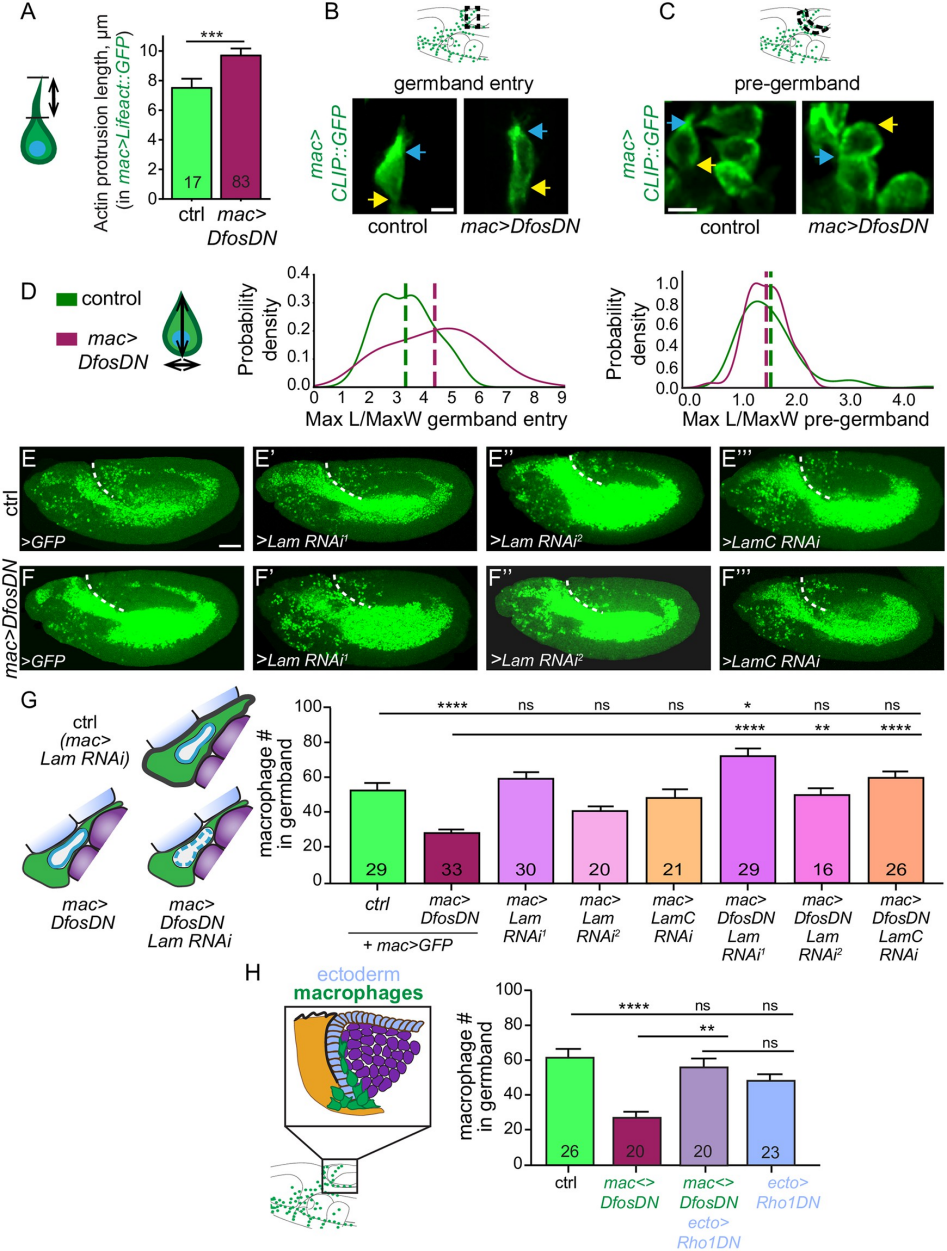
Dfos aids macrophage gb invasion against the resistance of surrounding tissues and buffers the nucleus. **(A)** Quantification from live embryos shows that the length of the F-actin protrusion of the first entering macrophage is longer in macrophages expressing DfosDN. *p* = 0.011. The F-actin protrusion labeled with *srpHemo-Gal4* driving *UAS-LifeAct*::*GFP* was measured in the direction of forward migration (see schematic). SD: 2.4, 3.7. **(B-C)** Stills from 2-photon movies of St 11 embryos showing **(B)** the first macrophages entering the gb and **(C)** macrophages in the pre-gb zone in the control and in a line expressing DfosDN in macrophages. Microtubules are labeled with *srpHemo-Gal4* driving *UAS-CLIP*::*GFP*. A blue arrow indicates the front and a yellow arrow indicates the rear of the macrophage. Schematics above indicate where images were acquired. **(D)** Schematic at left shows macrophage measurements: vertical line for the maximum length and horizontal line for the maximum width. Histograms show the probability density distributions of the aspect ratios (maximum length over maximum width) of the first macrophage entering the gb (left) and macrophages in the pre-gb (right). Macrophages expressing *DfosDN* are more elongated than the controls. Control vs. mac>*DfosDN* aspect ratios at gb entry *p* = 0.0011, in pre-gb *p* = 0.53. SD: in gb 1.0, 1.6; in pre-gb 0.5, 0.5. (**E-F”’**) Confocal images of St 12 embryos expressing RNAi against Lamin or LaminC in macrophages in **(E-E”’)** the control, or **(F-F”’)** in embryos also expressing DfosDN in macrophages. *srpHemo-GAL4* used as driver. *Lam RNAi*^*1*^: GD45636. *Lam RNAi*^*2*^: KK107419. *LamC RNAi*: TRiP JF01406. **(G)** Macrophage RNAi knockdown of Lamins, which can increase nuclear deformability did not affect macrophages numbers in the gb in the control. In embryos in which macrophages expressed DfosDN, Lamin knockdown rescued their reduced numbers in the gb. Control vs. *DfosDN p* < 0.0001. Control vs. *Lam RNAi*^*1*^ *p* > 0.99, vs. *Lam RNAi*^*2*^ *p* = 0.83, vs. *LamC RNAi p* > 0.99. Control vs. *DfosDN*, *Lam RNAi*^*1*^ *p* = 0.024, vs. *DfosDN*, *Lam RNAi*^*2*^ *p* > 0.99, vs. *DfosDN*, *LamC RNAi p* > 0.99. *DfosDN* vs. *DfosDN*, *Lam RNAi*^*1*^ *p* < 0.0001, vs. *DfosDN*, *Lam RNAi*^*2*^ *p* = 0.0049, vs. *DfosDN*, *LamC RNAi p* < 0.0001. SD: 22, 10, 19, 11, 21, 23, 16, 20. (**H**) Expressing *DfosDN* in macrophages reduces their number in the gb. Concomitantly reducing tissue tension in the ectoderm (light blue in schematic) through Rho1DN substantially rescues invasion. *srpHemo-QF QUAS* control (*mac<>)* governed macrophage expression and *e22c-GAL4* ectodermal (*ecto>*). Control vs. *mac<>DfosDN p* < 0.0001 (56% reduction), vs. *mac<>DfosDN; ecto>Rho1DN p* > 0.99, vs. *ecto>Rho1DN p* = 0.11. *mac<>DfosDN* vs. *mac<>DfosDN; ecto>Rho1DN p* < 0.0001, vs. *ecto>Rho1DN p* = 0.0044. *mac<>DfosDN; ecto>Rho1DN* vs. *ecto>Rho1DN p* > 0.99. SD: 23, 16, 21, 18. Macrophages are labeled in **B-C** by *srpHemo-Gal4* driving *UAS-CLIP*::*GFP*, and in **E-F’”** by *srpHemo-Gal4 UAS-mCherry*::*nls*. ****p < 0.0001, ***p < 0.005, **p < 0.01, *p < 0.05. Unpaired *t* test was used for **A**, one-way ANOVA with Tukey post hoc for **G-H**. The number shown within the column corresponds to measurements in **A**, and analyzed embryos in **G-H**. Scale bar 5 μm in **B-C**, and 50 μm in **E-F**”’. The data underlying the graphs can be found in S1 Data. ctrl, control; gb, germband; ns, not significant; RNAi, RNA interference.

We examined what consequence these lower cortical F-actin levels had on the cellular behavior of macrophages during entry. Quantitation showed that the actin protrusion that macrophages initially insert between the ectoderm and mesoderm during invasion was actually longer in the *mac>DfosDN>LifeAct*::*GFP* macrophages than in the control (Fig 5A and S5A Fig and S4 Movie). We then performed live imaging of macrophages labeled with CLIP::GFP to visualize microtubules and thus cell outlines in both genotypes; we determined the aspect ratio (maximal length over width) that the first entering cell displays as it enters into the gb. Unlike the control, the first DfosDN-expressing macrophage was extended even before it had fully moved its rear into the gb (S5B Fig). We carried out measurements, taking only the first cells that had entered the gb to be able to clearly distinguish the rear of the first macrophage from the tips of following cells (Fig 5B). We also avoided including in this measurement the forward protrusion and determined that the first DfosDN-expressing macrophage inside the gb displays an average increase of 23% in the maximal length (L) of the cell body and a 12% reduction in the maximal width (W) (Fig 5D and S5C Fig). Interestingly, in the pre-gb zone, the aspect ratio (max L/W) of *mac>DfosDN* macrophages was not different from control macrophages (Fig 5C and 5D), although the *mac>DfosDN* cells were 9% smaller in both their length and width (S5D Fig). This suggested that the gb could impose resistance on the entering macrophage, an effect that *mac>DfosDN* macrophages have trouble overcoming due to their compromised cortical actin cytoskeleton.

### Dfos promotes advancement of macrophages against the resistance of the surrounding tissues and buffers the nucleus

We therefore examined how the properties of the gb tissues and macrophages interact during invasion. We first investigated if the macrophage nucleus impedes normal invasion by varying levels of the 2 *Drosophila* Lamin genes, Lam and LamC, both equally related to the vertebrate lamins A and B1 [51] and both shown to affect nuclear stiffness and deformability [52, 53]. Overexpressing Lam (S5E Fig) or knocking down either of these Lamins in macrophages through RNAi [54] did not change macrophage numbers in the gb of wild-type embryos (Fig 5E–5E”’ and 5G), suggesting that the properties of the macrophage nucleus are not a rate-limiting parameter during normal tissue invasion into the narrow path between the ectoderm and mesoderm. This result also argues that Lamins’ capacity to alter gene expression is not normally important for invasion [55]. However, in *mac>DfosDN* macrophages, knockdown of these Lamins was able to rescue the gb invasion defect (Fig 5E–G), supporting the conclusion that the properties of the nucleus affect invasion in the absence of the higher levels of cortical actin Dfos normally induces. To directly test if reducing the tension of surrounding tissues can counteract the absence of Dfos, we expressed Rho1DN in the ectoderm with the *e22c-GAL4* driver while expressing *QUAS-DfosDN* in macrophages with the GAL4-independent Q-system driver we had constructed, *srpHemo-QF2* [33]. Rho1 through ROCK is a key regulator of Myosin activity, epithelial tension, and tissue stiffness [56,57]; Myosin II is essential for actin contractility [58] and tension in the *Drosophila* gb ectoderm [16]. Indeed, we found that this reduction of ectodermal tension substantially rescued DfosDN expressing macrophage numbers in the gb (Fig 5H). Taken together, our results argue that Dfos aids *Drosophila* macrophages in withstanding the resisting force of surrounding cells against the nucleus during invasion into tissues.

## Discussion

**Fig 6:**
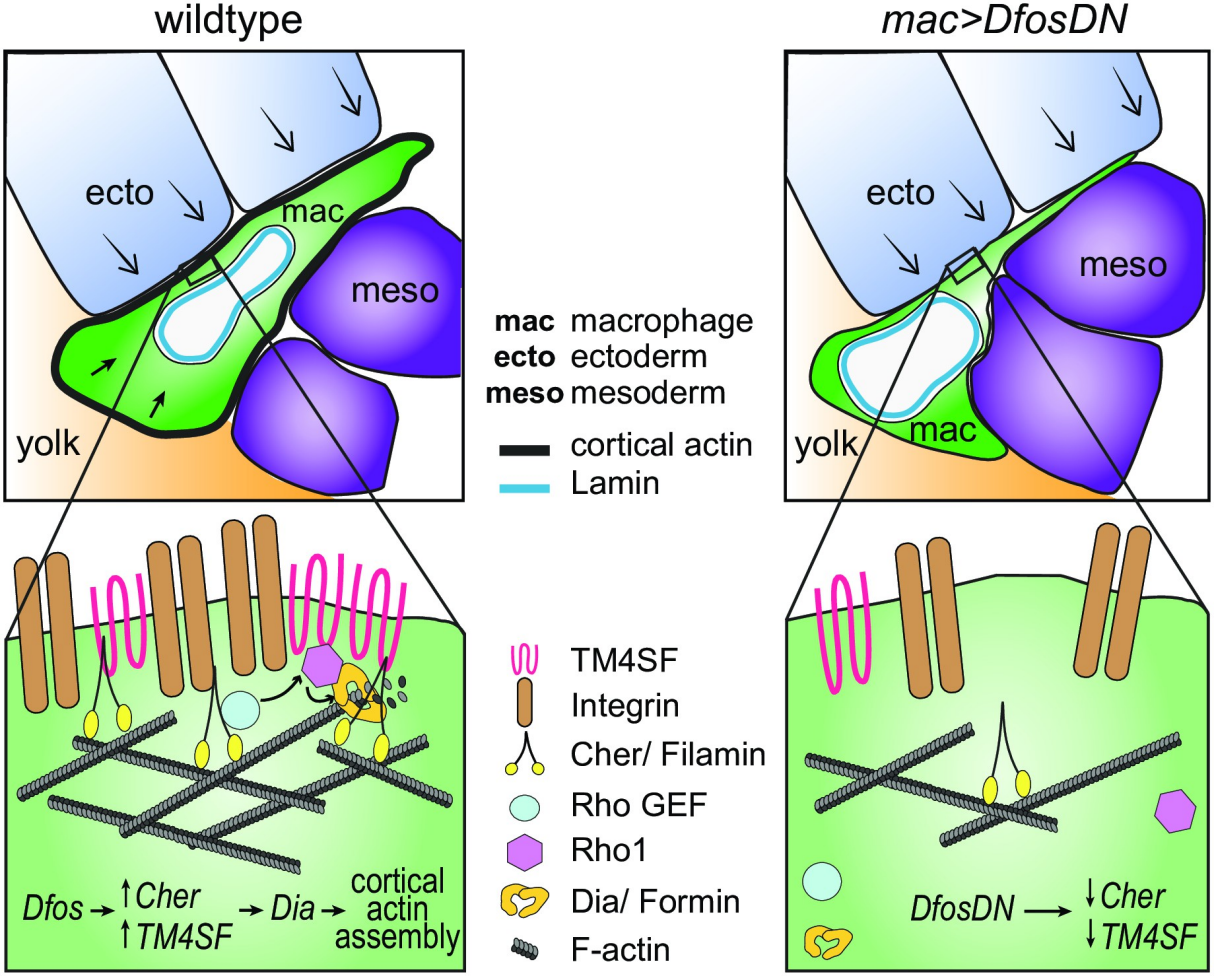
Model: Dfos increases actin assembly and cross-linking through the tetraspanin TM4SF and the filamin Cher to counter surrounding tissue resistance. We propose a speculative model for how Dfos tunes the cortical actin properties of *Drosophila* embryonic macrophages to aid their infiltration against the resistance of the surrounding gb tissue. We have shown that Dfos leads to an increase of the tetraspanin TM4SF and the filamin Cher. Filamins cross-link actin and have been shown to bind to RhoGEFs; Tetraspanins bind to Integrins, Rho GTPases, and Filamins in other systems (see S6 Fig). Thus, we hypothesize that in *Drosophila* macrophages, TM4SF and the filamin Cher could form a network at the cell surface of Integrin, actin, and upstream signaling molecules, recruiting Rho GEFs and leading to the activation of Rho1 GTPase and the actin polymerizing Formin Dia. Dia activation could occur through direct binding to active Rho1 and through direct interaction with TM4SF or Cher. Validation in *Drosophila* of all the protein interactions we propose awaits biochemical analysis. Through this pathway, a more cross-linked and dense F-actin network would form, aiding the macrophage in moving its cell body into the ecto–meso interface. The presence of Lamin around the nuclear membrane would not normally affect this process since the dense cross-linked cortical actin network would help macrophages withstand the load of the surrounding tissues. However, in the DfosDN-expressing macrophages, the loss of Cher and TM4SF would lead to reduced cross-linked actin levels at the cell cortex, making the stiffness of the nucleus the rate limiting step for macrophage infiltration of the gb tissue. Cher, Cheerio; ecto, ectoderm; gb, germband; mac, macrophage; meso, mesoderm.

We identify the ability to tune the state of the cortical actin cytoskeleton as a key capacity for immune cells migrating into and within tissue barriers in vivo. We find that macrophages up-regulate a program governed by the transcription factor Dfos to enable this. Dfos in *Drosophila* is known to regulate the movement during dorsal or wound closure of epithelial sheets [29,30,59,60] as well as the development of epithelial tumors and their dissemination [61–64]. Here, we define a different role, namely that Dfos enables a stream of individual immune cells to efficiently push their way into tissues, a process that is aided rather than hampered by the presence of the ECM [18,19]. This function appears to be specifically required for invasion as we observe no defects in *DfosDN* macrophages’ migratory speed in open environments. *DfosDN* macrophages display decreased actin at the cell circumference and an elongated shape within the confinement of the gb, suggesting a defect in the stiffness of the cortex. Strikingly, only in the presence of *DfosDN* does the state of the nucleus become relevant, with reductions in lamins shown to underlie nuclear stiffness [52] enhancing the ability of macrophages to invade. These findings along with the ability of a softened ectoderm to substantially rescue the *DfosDN* macrophages’ gb invasion defect lead us to propose the model (Fig 6) that Dfos permits efficient initial translocation of the macrophage body under ectodermal reactive load by forming a stiff cortical actin shell that counteracts surrounding tissue resistance and protects the nucleus from undergoing high levels of mechanical stress during tissue entry.

### A molecular program for tissue invasion that strengthens cortical actin

Crucial mediators of this process are 2 actin regulators, the filamin Cher, known to be a Dfos target in epithelia, and the previously uncharacterized membrane scaffold tetraspanin TM4SF. We show that both require Dfos for higher mRNA levels in macrophages and present correlative evidence that these classes of genes are also up-regulated by vertebrate c-fos. Each of these Dfos targets is required for macrophage invasion; overexpression of TM4SF in macrophages can rescue the *DfosDN* tissue invasion phenotype. We propose that these targets act together to strengthen the actin cytoskeleton for tissue invasion. Higher Filamin levels cross-link actin filaments into resilient and stiffer networks maintaining cell integrity during mechanical stress [65–67]. This aids the distribution of forces from focal adhesions (FAs) across the entire migrating cell body, since Filamins can bind directly to Integrin, and do so even more strongly under strain [35,68–70]. Tetraspanins, self-associating multipass transmembrane proteins, also can bind Integrin, forming microdomains of adhesion molecules, receptors, and their intracellular signaling complexes, including Rho GTPases [71–76]. Filamins similarly bind receptors, regulators of actin assembly, Rho GTPases, and the Rho GEFs Trio and Vav2 [47,48,77–80]. We observe reduced cortical levels of F-actin, active Rho1, and the actin polymerizing formin Diaphanous in the absence of either Dfos, the filamin Cher, or the tetraspanin TM4SF. Thus, our data support the hypothesis that these Dfos targets enhance the cortical recruitment and activation of the formin Dia to stimulate actin polymerization at least in part through the recruitment of RhoGEFs, which enhance GTP-bound Rho1, which can activate Dia (Fig 6 and S6 Fig) [44,45,81–84]. Cher and/or TM4SF may also directly contribute to Dia activation, as Rho-independent mechanisms of activation have been proposed [42] and direct binding between Filamins and Formins has been observed [85,86]. Full confirmation of our hypotheses requires future biochemical characterization of the interactions of these players in *Drosophila*. Dfos’ up-regulation of Cher and TM4SF could thus lead to a supranetwork in which ECM-anchored FAs connect to a strong cross-linked cortical actin lattice, allowing Myosin contraction to be converted into cellular advancement despite resistance from the flanking ectoderm.

We demonstrate that the actin nucleating formin Dia is important for *Drosophila* macrophage invasion and capable of rescuing the defects in the *DfosDN* mutant. Unlike the formin Ena, which mediates chemotaxis [40], Dia is not required for general *Drosophila* macrophage migration and instead allows macrophages to recoil away from one another [41]. Dia could be required for macrophages specifically when they face resistance from their surroundings and need to increase their cortical tension. Modeling indicates that Dia1’s regulation of cortical tension requires an optimal combination of actin cross-linking and intermediate actin filament length [87]. *Drosophila* Dia is a more processive nucleator than Ena [88] and thus could create the intermediate length actin filaments that enable higher levels of macrophage cortical tension and strain stiffening [89] on all sides of the cell during their invasion.

Our findings thus demonstrate that there are commonalities in the molecular mechanisms by which *Drosophila* cells invade into either confluent tissues or the ECM. Dfos’s up-regulation of the filamin Cher is also required in tumor cells and aneuploid epithelial cells to enhance ECM breaching [61,63]. Both cell types displayed enhanced levels of cortical filamentous actin, which in the tumors is concomitant with Dia up-regulation [63]. In the oocyte, Filamin is required for follicle cell intercalation, and border cells display higher levels of Filamin and F-actin to maintain cellular integrity during migration between nurse cells [90,91]. The mediator of these increased F-actin levels, MAL-D, can be activated by Dia [91]. Thus, while MMPs may be specific to ECM crossing, a denser and more cross-linked actin cortex due to increased levels of the filamin Cher and activity of the formin Dia could be a common feature of *Drosophila* cells moving through the resistance of either ECM or surrounding tissues. Determining if such shifts in cell surface actin properties underlie some vertebrate cancer cells’ capacity to metastasize even in the presence of MMP inhibitors is an interesting area of inquiry [92,93].

### Implications for vertebrate immune cell migration

Our work also suggests a new perspective on the migration of some vertebrate immune cells. We find that altering lamin levels does not normally affect *Drosophila* macrophage tissue invasion. This contrasts with results showing that nuclear deformability from lower lamin levels underlies the migration of some immune cell types through narrow constrictions engineered from rigid materials [94,95]. However, negotiation of such extremely challenging in vitro environments can lead to DNA damage [96], and higher nuclear flexibility caused by lower lamin levels is associated with increased cell death [97]. A robust cell surface actin layer could allow long-lived cells or those not easily replenished to protect their genome as they move through resistant yet deformable environments. Embryonic *Drosophila* and vertebrate tissue-resident macrophages migrate into tissues during development, survive into the adult, and serve as founders of proliferative hematopoetic niches [3,4,98–101]. Tissue-resident memory T cells migrate in response to infection in mature animals, are long lived, and are not easily renewed from the blood [102]. Thus, the importance of nuclear mechanics for migration in challenging in vivo environments should be explored for a broader range of immune cells as well as the utilization of cortical actin as a strategy for genomic protection.

## Materials and methods

### Fly strains and genetics

Flies were raised on standard food bought from IMBA (Vienna, Austria) containing agar, cornmeal, and molasses with the addition of 1.5% Nipagin. Adults were placed in cages in a fly room or a Percival DR36VL incubator maintained at 25°C and 65% humidity or a Sanyo MIR-153 incubator at 29°C within the humidity controlled 25°C fly room; embryos were collected on standard plates prepared in house from apple juice, sugar, agar, and Nipagin supplemented with yeast from Lesaffre (Marcq, France) on the plate surface. Fly crosses and embryo collections for RNAi experiments (7-hour collection) as well as live imaging (6-hour collection) were conducted at 29°C to optimize expression under GAL4 driver control [103]. All fly lines utilized are listed below.

### Fly stocks

*srpHemo-GAL4 (mac>)* was provided by K. Brückner (UCSF, USA) [9]. *Oregon R (control)*, *P{CaryP}attP2 (control)*, *P{CaryP}attP40 (control)*, *kay*^*2*^ *(Dfos*^*2*^*)*, *(UAS-Fra)2 (Dfos)*, *UAS-Rho1*.*N19 Rho1DN)*, *UAS-fbz (DfosDN)*, *UAS-kayak* RNAi *(Dfos RNAi)* TRiP HMS00254 and TRiP JF02804, *UAS-dia RNAi* TRiP HM05027, *UAS-LamC RNAi* TRiP JF01406 and TRiP HMS00308, *e22c-GAL4 (ecto>)*, *Resille*::*GFP*, *UAS-GFP*::*nls*, *UAS-dia*::*EGFP*, *UAS-diaRBD*::*EGFP*, *UAS-mCherry*::*nls*, *UAS-CD8*::*GFP* lines were obtained from the Bloomington Stock Center (Indiana, USA). *kay*^*1*^ (*Dfos*^*1*^*)* line was provided by O. Schuldiner (WIS, Israel). *UAS-dia*::*deltaDad*::*EGFP (diaCA)* and *srpHemo-GAL4 UAS-CLIP*::*GFP (mac>CLIP*::*GFP)* lines were provided by B. Stramer (KCL, UK). *UAS-cher*::*FLAG (cher)* line was provided by M. Uhlirova (CECAD, Germany). *w[1118] (control)*, *UAS-cher* RNAi KK107451, *UAS-TM4SF* RNAi KK102206, *UAS-Lam RNAi*^*1*^ GD45636, *UAS-Lam RNAi*^*2*^ KK107419 lines were obtained from the Vienna *Drosophila* Resource Center (Austria).

### Extended genotypes

Here, we list the lines used in each figure; we state first the name from FlyBase; in parentheses, the name used in the figure panels is provided.

#### Fig 1 and S1 Fig

Fig 1D: *Oregon R*. Fig 1E–1G: *srpHemo-GAL4*, *UAS-GFP (control)*. S1A and S1F Fig: *srpHemo-Gal4*, *srpHemo-H2A*::*3xmCherry/P{CaryP}attP2 (control)*. Fig 1H: *srpHemo-GAL4*, *UAS-GFP; kay*^*1*^ *(Dfos*^*1*^*)*. Fig 1I–1L and S1B and S1G Fig: *srpHemo-GAL4*, *UAS-GFP*::*nls/+ (control 1)*. Fig 1H, 1J and 1N: *srpHemo-GAL4*, *UAS-GFP/+; kay*^*1*^ *(Dfos*^*1*^*)*. Fig 1K and 1N and S1B Fig: *srpHemo-GAL4*, *UAS-GFP*::*nls/+; kay*^*2*^ *(Dfos*^*2*^*)*. Fig 1L and 1N: *srpHemo-GAL4*, *UAS-GFP*::*nls/(UAS-Fra)2; kay*^*2*^ *(Dfos*^*2*^;*mac>Dfos)*. Fig 1O and 1Q: *10XUAS-IVS-myr*::*GFP/+; srpHemo-Gal4*, *srpHemo-H2A*::*3xmCherry/+ (control 2 and control)*. Fig 1P and 1Q: *UAS-DfosDN/+; srpHemo-Gal4*, *srpHemo-H2A*::*3xmCherry/+ (mac>DfosDN)*. S1C and S1F Fig: *srpHemo-Gal4*, *srpHemo-H2A*::*3xmCherry/ UAS GFP*::*nls (ctrl)*. *srpHemo-Gal4*, *srpHemo-H2A*::*3xmCherry/UAS-fbz (mac>DfosDN)*. S1D Fig: *srpHemo-Gal4*, *srpHemo-H2A*::*3xmCherry /+ (ctrl)*. *srpHemo-Gal4*, *srpHemo-H2A*::*3xmCherry/UAS-DfosDN (mac>DfosDN)*. Fig 1R and S1E, S1G and S1I Fig: *UAS-GFP; srpHemo-Gal4*, *srpHemo-H2A*::*3xmCherry (ctrl)*. *UAS-Dfos RNAi HMS00254/srpHemo-Gal4*, *srpHemo-H2A*::*3xmCherry (mac>DfosRNAi*^*1*^*)*. *UAS-Dfos RNAi JF02804/srpHemo-Gal4*, *srpHemo-H2A*::*3xmCherry (mac>DfosRNAi*^*2*^*)*. S1H Fig: *srpHemo-GAL4*, *UAS-GFP*::*nls/+ or /(UAS-Fra)2 (mac>Dfos)*. S1I Fig: *UAS-GFP; UAS-Dfos RNAi HMS00254/ srpHemo-Gal4*, *srpHemo-H2A*::*3xmCherry (mac>DfosRNAi*^*1*^*+ GFP)*. *UAS-GFP; UAS-Dfos RNAi JF02804/srpHemo-Gal4*, *srpHemo-H2A*::*3xmCherry (mac>DfosRNAi*^*2*^*+ GFP)*.

#### Fig 2 and S2 Fig

Fig 2A and 2C–2I and S2A, S2B and S2E Fig: srpHemo-Gal4, *srpHemo-H2A*::*3xmCherry*/+ (control). Fig 2D: *srpHemo-Gal4*, *srpHemo-H2A*::*3xmCherry/*+ (3 movies) and *Resille*::*GFP/+; srpHemo-Gal4*, *srpHemo-H2A*::*3xmCherry/*+ (4 movies, control) and *Resille*::*GFP/+; srpHemo-Gal4*, *srpHemo-H2A*::*3xmCherry*/+ (3 movies) and *Resille*::*GFP/+; srpHemo-Gal4*, *srpHemo-H2A*::*3xmCherry/UAS-DfosDN* (4 movies, DfosDN). Fig 2A and 2C–2I and S2A, S2B and S2E Fig: *srpHemo-Gal4*, *srpHemo-H2A*::*3xmCherry/UAS-fbz (mac>DfosDN)*. S2C and S2D Fig: *srpHemo-GAL4*, *UAS-GFP*.*nls/+ (control)*. S2C and S2D Fig: *srpHemo-GAL4*, *UAS-GFP*.*nls/+; kay*^*2*^ *(Dfos*^*2*^*)*.

#### Fig 3 and S3 Fig

Fig 3C and 3G and S3D Fig: *UAS-Dicer2;; srpHemo-Gal4*, *srpHemo*-*H2A*::3x*mCherry/w*^*1118*^ (control). Fig 3D and 3G and S3D Fig: *UAS-Dicer2; UAS-TM4SF RNAi* KK10220/*+; srpHemo-Gal4*, *srpHemo*-*H2A*::3x*mCherry/+* (*mac>TM4SF RNAi*). Fig 3E and 3G and S3D Fig: *UAS-Dicer2; UAS-cher RNAi* KK107451*/+; srpHemo-Gal4*, *srpHemo*-*H2A*::3x*mCherry/+* (*mac>cher RNAi*). Fig 3F and 3G: *UAS-Dicer2; UAS-cher RNAi* KK107451/*UAS-TM4SF RNAi* KK102206; *srpHemo-Gal4*, *srpHemo*-*H2A*::3x*mCherry/+* (*mac>TM4SF RNAi*, *cher RNAi*). Fig 3H and 3L: *srpHemo-GAL4*, *UAS-mCherry*::*nls/UAS-mCD8*::*GFP* (control). Fig 3I and 3L: *srpHemo-GAL4*, *UAS-mCherry*::*nls/UAS-mCD8*::*GFP; UAS-fbz/+* (*mac>DfosDN*). Fig 3J and 3L: *srpHemo-GAL4*,*UAS-mCherry*::*nls/UAS-cheerio*::*FLAG; UAS-fbz/+* (*mac>DfosDN*, *cher*). Fig 3K and 3L: *srpHemo-GAL4*,*UAS-mCherry*.*nls/UAS-TM4SF; UAS-fbz/+* (*mac>DfosDN*, *TM4SF*). Fig 3L: *srpHemo-GAL4*, *UAS-mCherry*::*nls/ UAS-TM4SF* (*mac>TM4SF*). Fig 3L: *srpHemo-GAL4*, *UAS-mCherry*::*nls/UAS-cher* (*mac>cher*). S3A–S3C Fig: *srpHemo-Gal4*, *srpHemo*-3x*mCherry/+* (control). S3A–S3C Fig: *srpHemo-Gal4*, *srpHemo*-3x*mCherry/UAS-fbz* (*mac>DfosDN*).

#### Fig 4 and S4 Fig

Fig 4A: *srpHemo-3xmCherry; kay*^*1*^ (*Dfos*^*1*^) *and srpHemo-3xmCherry; +*. Fig 4B and 4D, and S4A Fig: *srpHemo-Gal4*, *srpHemo*-*moe*::3x*mCherry/+;UAS-mCD8*::*GFP/+*(*Control*). Fig 4C and 4D and S4A Fig: *srpHemo-Gal4*, *srpHemo*-*moe*::3x*mCherry/UAS-fbz* (*mac>DfosDN*). S4A Fig: *w*^*118*^. Fig 4E and 4F: *srpHemo-Gal4*, *srpHemo*-*moe*::3x*mCherry/w*^*118*^ (*Control*). Fig 4E and 4G: *srpHemo-Gal4*, *srpHemo*-*moe*::3x*mCherry/UAS-cher RNAi* KK107451 (*mac>cher RNAi*). Fig 4E and 4H: *srpHemo-Gal4*, *srpHemo*-*moe*::3x*mCherry/UAS-TM4SF RNAi* KK102206 (*mac>TM4SF RNAi*). Fig 4I and 4J: *srpHemo-GAL4*, *UAS-mCherry*.*nls/UAS-mCD8*::*GFP* (*control*). Fig 4I’, J: *srpHemo-GAL4*, *UAS-mCherry*.*nls/UAS-DiaΔDad*::*EGFP; UAS-fbz/+* (*mac>DfosDN*, *diaCA*). Fig 4J: *srpHemo-GAL4*, *UAS-mCherry*.*nls/UAS-mCD8*::*GFP; UAS-fbz/+ (mac>DfosDN)*. Fig 4J: *srpHemo-GAL4*, *UAS-mCherry*.*nls/ UAS-DiaΔDad*::*EGFP (mac>diaCA)*. S4B Fig: #1: *UAS-GFPnls; srpHemo-Gal4*, *srpHemo-H2A*::*3xmCherry*. #2: *UAS-GFPnls*/*srpHemo-Gal4*, *srpHemo-H2A*::*3xmCherry*; *Dfos*^*1*^. #3: *UAS-GFPnls*/ *srpHemo-Gal4*, *srpHemo-H2A*::*3xmCherry*; *Dfos*^*2*^. #4: *UAS-DiaΔDad*::*EGFP*/*srpHemo-Gal4*, *srpHemo-H2A*::*3xmCherry*; *Dfos*^*1*^. #5: *UAS-DiaΔDad*::*EGFP*/*srpHemo-Gal4*, *srpHemo-H2A*::*3xmCherry*; *Dfos*^*2*^. Fig 4K and 4L and S4C and S4D Fig: *UAS-Dicer2;; srpHemo-Gal4*, *srpHemo*-*H2A*::3x*mCherry/P{CaryP}attP40* (control). Fig 4K’ and 4L and S4C and S4D Fig: *UAS-Dicer2;+; srpHemo-Gal4*, *srpHemo*-*H2A*::3x*mCherry/ UAS-dia RNAi* HM05027 (*mac>dia RNAi*^*1*^). Fig 4L and S4C and S4D Fig: *UAS-Dicer2;+; srpHemo-Gal4*, *srpHemo*-*H2A*::3x*mCherry/UAS-dia RNAi* HMS00308 (*mac>dia RNAi*^*2*^). Fig 4M and 4N and S4E Fig: (control) *UAS-dia*::*EGFP/+; UAS-nlacz/ srpHemo-Gal4*, *10XUAS-IVS-myr*::*tdTomato*. *UAS-dia*::*EGFP/+; UAS-Rho1N*.*19)/srpHemo-Gal4*, *10XUAS-IVS-myr*::*tdTomato* (*mac>Rho1DN*). *UAS-dia*::*EGFP/+; UAS-fbz/srpHemo-Gal4*, *10XUAS-IVS-myr*::*tdTomato* (*mac>DfosDN*). *UAS-dia*::*EGFP/+; UAS-cher RNAi KK107451/srpHemo-Gal4*, *10XUAS-IVS-myr*::*tdTomato* (*mac>cher RNAi*). *UAS-dia*::*EGFP/+; UAS-TM4SF RNAi KK102206/srpHemo-Gal4*, *10XUAS-IVS-myr*::*tdTomato* (*mac>TM4SF RNAi*). Fig 4O and 4P and S4F Fig: *UAS-diaRBD*::*GFP/+; srpHemo-Gal4*, *10XUAS-IVS-myr*::*tdTomato/UAS-nlacZ* (control). *UAS-diaRBD*::*GFP/+; srpHemo-Gal4*, *10XUAS-IVS-myr*::*tdTomato/UAS-Rho1N*.*19* (*mac>Rho1DN*). *UAS-diaRBD*::*GFP/UAS-fbz; srpHemo-Gal4*, *10XUAS-IVS-myr*::*tdTomato/+* (*mac>DfosDN*). *UAS-diaRBD*::*GFP/UAS-cher RNAi KK107451; srpHemo-Gal4*, *10XUAS-IVS-myr*::*tdTomato/+* (*mac>cher RNAi*). *UAS-diaRBD*::*GFP/UAS-TM4SF RNAi KK102206; srpHemo-Gal4*, *10XUAS-IVS-myr*::*tdTomato/+* (*mac>TM4SF RNAi*).

#### Fig 5 and S5 Fig

Fig 5A and S5A Fig: *srpHemo-Gal4 UAS-LifeActGFP UAS-RedStinger* (control); *srpHemo-Gal4 UAS-LifeActGFP UAS-RedStinger; UAS-DfosDN* (*mac>DfosDN*). Fig 5B–5D and S5B–S5D Fig: *srpHemo-Gal4*, *UAS-CLIP*::*GFP*, *UAS-RedStinger* (control). Fig 5B–5D and S5B–S5D Fig: *srpHemo-Gal4*, *UAS-CLIP*::*GFP*, *UAS-RedStinger; UAS-fbz* (*mac>DfosDN*). Fig 5E and 5G: *srpHemo-GAL4*, *UAS-mCherry*.*nls/UAS-mCD8*::*GFP* (control). Fig 5E’, 5E” and 5G: *srpHemo-GAL4*, *UAS-mCherry*.*nls/UAS-Lamin RNAi* GD45636, KK107419 (*mac>Lam RNAi*^1^ and *mac>Lam RNAi*^2^, respectively). Fig 5E”’ and 5G: *srpHemo-GAL4*, *UAS-mCherry.nls/UAS-LaminC RNAi* TRIP JF01406 (*mac>LamC RNAi*). Fig 5F and 5G: *srpHemo-GAL4, UAS-mCherry.nls/UAS-mCD8*::*GFP; UAS-fbz/+* (*mac>DfosDN*). Fig 5F’, 5F” and 5G: *srpHemo-GAL4, UAS-mCherry.nls/UAS-Lam RNAi* (*Lam RNAi*^1^ = GD45636, *Lam RNAi*^2^ = KK107419); *UAS-fbz/+* (*mac>DfosDN, Lam RNAi*^1^ and *mac>DfosDN, Lam RNAi*^2^). Fig 5F”’ and 5G: *srpHemo-GAL4, UAS-mCherry.nls/UAS-LaminC RNAi* TRIP JF01406; *UAS-fbz/+* (*mac>DfosDN, LamC RNAi*). Fig 5H: *e22c-Gal4,srpHemo-H2A*::*3xmCherry/+* (control). Fig 5H: *srpHemo-QF/ srpHemo-H2A*::*3xmCherry; QUAS-fbz/UAS-Rho1.N12* (*mac<>DfosDN*). Fig 5H: *e22c-Gal4, srpHemo-H2A*::*3xmCherry/srpHemo-QF; +/ UAS-Rho1.N12* (*ecto>Rho1DN*). Fig 5H: *srpHemo-QF/ e22c-Gal4, srpHemo-H2A*::*3xmCherry; UAS-Rho1.N12/QUAS-fbz* (*mac<>DfosDN, ecto>rho1DN*). S5E Fig: *+;UAS-GFP*::*nls, srpHemo-GAL4* (control). *+;UAS-GFP*::*Lamin, srpHemo-GAL4* (*mac>Lam*).

### Cloning and generation of QUAS-DfosDN line

The fragment was amplified from genomic DNA of the published *UAS-fbz (UAS-Dfos DN)* line [31] using primers encompassing a 5′ consensus translation initiation sequence followed by the bZIP fragment and containing BglII and XhoI restriction sites: 5′-GAAGATCTATTGGGAATTCAACATGACCCCG-3′ and 5′-CCCTCGAGTCAGGTGACCACGCTCAGCAT-3′. The resulting fragment was cloned into the pQUASt vector, a gift from Christopher Potter (Addgene plasmid #104880). The final construct was sequenced and injected into the attP2 landing site by BestGene (Chino Hills, CA, USA).

### Cloning and generation of UAS-TM4SF line

The TM4SF open reading frame was amplified from the DGRC GH07902 cDNA clone (#3260, Fbcl0121651), using primers acagcgGAATTCATGGCATTGCCGAAGAAAAT and acagcgTCTAGATTAAAAGCTAATCGTCTGTCATT. The PCR product and the pUASt-aTTB vector (DGRC plasmid #1419) were digested with EcoRI and XbaI, and ligated. After sequencing, the construct was injected into the landing site line, (*y*^*1*^ *M{vas-int*.Dm}ZH-2A w*; *M{3xP3-RFP*.*attP}ZH-51D*, BL 24483), to produce second chromosome inserts. All male survivors were crossed to *w*; *Sp/CyO*; *PrDr/TM3Ser* virgins. Transformants were recognized by eye color and crossed again to *w*; *Sp/CyO*; *PrDr/TM3Ser* virgins to get rid of the X chromosomal integrase.

### Embryo staging

Laterally oriented embryos with complete gb extension and the presence of stomadeal invagination were staged based on gb retraction from the anterior as a percentage of total embryo length. Embryos with no gb retraction were classified as Stage 11, 30% retraction early Stage 12, 60% retraction Stage 12, and 70% Stage 13. Imaged embryos are shown throughout paper in a lateral orientation with anterior to the left and dorsal up.

### In situ hybridization and immunofluorescence

Embryos were dechorionated by 5-minute treatment with 50% Chlorox bleach. After extensive washing with water, embryos were fixed with 3.7% formaldehyde/heptane for 20 minutes followed by methanol devitellinization for in situ hybridization and visualization of 3xmCherry or tdTomato. The *Dfos* cDNA clone SD04477 was obtained from the DGRC. T7 or T3 polymerase-synthesized digoxigenin-labeled antisense probe preparation and in situ hybridization was performed using standard methods [104]. Images were taken with a Nikon-Eclipse Wide field microscope with a 20X 0.5 NA DIC water Immersion Objective. Fixed embryos were blocked in BBT (0.1 M PBS + 0.1% TritonX-100 + 0.1% BSA) for 2 hours at RT and then incubated with antibodies overnight at 4°C. Antibodies were used at the following dilutions: Rabbit anti-Dfos 1:50 (Julia Zeitlinger (Stowers)), mouse anti α-GFP 1:500 (Abcam, Cambridge, UK, ab13970), goat anti-mCherry 1:200 (Invitrogen| ThermoFisher Scientific, Waltham, MA). Afterwards, embryos were washed in BBT for 2 hours, incubated with secondary antibodies at RT for 2 hours, and washed again for 2 hours. Secondary antibodies and Phalloidin were used at the following dilutions: anti-mouse 488 1:500 or anti-mouse 633 1:200, anti-rabbit 488 1:300, and Phalloidin 1:300 (all from ThermoFisher Scientific, Waltham, MA, USA). Embryos were mounted overnight at 4°C in Vectashield Mounting Medium (Vector Labs, Burlingame, USA) and imaged with a Zeiss Inverted LSM700 and LSM800 Confocal Microscope using a Plain-Apochromat 20X/0.8 Air Objective or a Plain-Apochromat 63X/1.4 Oil Objective as required.

### Dfos antibody

The Dfos rabbit polyclonal antibody was produced for the lab of Julia Zeitlinger. It was raised by Genescript (Piscataway, NJ, USA) against the C-terminal end of *Drosophila* Kayak found in all isoforms and was purified against an N terminally His-tagged antigen corresponding to aa 73 to 595 of Kay isoform A. The internal Genescript order number is 163185–30, and in the Zeitlinger lab is referred to as anti-Kay/Fos Ab.

### Western

Cages were prefed on fresh yeast plates for 2 days. Late stage 11/early stage 12 embryos were handpicked using a Leica M205 fluorescent microscope on ice-cold apple juice plates. They were transferred to RIPA buffer (50 mM Tris, 150 mM NaCl, 1% NP-40, 1 mM EDTA, 0.5% Na-Deoxycholate, 0.1% SDS) with a Halt Protease/Phosphatase inhibitor cocktail (ThermoFisher, #78440) and lysed. After a 30-minute incubation on ice, they were centrifuged 15 minutes at 4°C at 15,000*g*. Then 10 μg of the cleared lysate were separated by SDS-PAGE using 4% to 15% Mini-PROTEAN TGX Precast Protein gels (Bio-Rad, #4561085) and blotted onto a Amersham Protran Premium western blotting nitrocellulose membrane (Sigma, #GE10600003). The nitrocellulose membrane was blocked with Pierce Clear Milk blocking buffer (ThermoFisher, #37587) and incubated in blocking buffer with anti-mCherry (Novus Biologicals, #NBP1-96752) at 1:1,000, and anti-Profilin (DSHB, #chi 1J) [105] at 1:50 antibodies over night at 4°C. The membrane was washed 3 times for 10 minutes with 1× PBS and incubated with Goat Anti-Mouse IgG (H+L)-HRP Conjugate (BioRad, #172–1011). Chemiluminescence was induced by incubation with SuperSignal West Femto Maximum Sensitivity Substrate (ThermoFisher, #34096) and recorded with a ChemieDoc MP (BioRad) molecular imager. Densitometric quantification of bands was done with ImageJ.

### Time-lapse imaging

Embryos were dechorionated in 50% bleach for 5 minutes, washed with water, and mounted in halocarbon oil 27 (Sigma) on a 24 × 50 mm high precision coverslip (Marienfeld Laboratory Glassware, No. 1.5H) between 2 bridges (approximately 0.5 cm high) of coverslips glued on top of each other or mounted in halocarbon oil 27 (Sigma) between an 18 × 18 mm coverslip (Marienfeld Laboratory Glassware, No. 1.5H) and an oxygen permeable membrane (YSI). The embryo was imaged on an upright multiphoton microscope (TrimScope, LaVision) equipped with a W Plan-Apochromat 40X/1.4 oil immersion objective (Olympus). GFP and mCherry were imaged at 860 nm and 1,100 nm excitation wavelengths, respectively, using a Ti-Sapphire femtosecond laser system (Coherent Chameleon Ultra) combined with optical parametric oscillator technology (Coherent Chameleon Compact OPO). Excitation intensity profiles were adjusted to tissue penetration depth and Z-sectioning for imaging was set at 1 μm for tracking. For long-term imaging, movies were acquired for 60 to 150 minutes with a frame rate of 25 to 45 seconds. A temperature control unit set to 29°C was utilized for all genotypes except *Dfos*^*2*^ for which the setting was 25°C.

### Image analysis

#### Macrophage cell counts

Autofluorescence of the embryo revealed the position of the gb for staging of fixed samples. Embryos with 40% (±5%) gb retraction (Stage 12) were analyzed for macrophage numbers in the pre-gb, within the gb, along the vnc, and in the whole embryo. For the *Dfos RNAi*, embryos with 70% gb retraction (Stage 13) were used for vnc counts. The pre-gb zone was defined based on embryo and yolk autofluorescence as an area on the yolk sac underneath the amnioserosa with borders defined posteriorly by the gb ectoderm and anteriorly by the head. Macrophages were visualized using confocal microscopy with a Z-stack step size of 2 μm, and macrophage numbers within the gb or the segments of the vnc were calculated in individual slices (and then aggregated) using the Cell Counter plugin in FIJI. Total macrophage numbers were obtained using Imaris (Bitplane) by detecting all the macrophage nuclei as spots.

#### Macrophage tracking, speed, persistence, mode of migration, and macrophage gb crossing analysis

Embryos with macrophage nuclei labeled with *srpHemo-H2A*::*3xmCherry* and the surrounding tissues with *Resille*::*GFP*, or with only macrophages labeled by *srpHemo-H2A*::*3xmCherry*, *or srpHemo>GFP*::*nls* were imaged, and 250 × 250 × 40 μm^3^ 3D stacks were typically acquired with approximately 0.2 × 0.2 × 1 μm^3^ voxel size every 39 to 41 seconds for approximately 2 hours. For imaging macrophages on vnc, frames were acquired at every 40 to 43 seconds for 30 minutes after macrophages started spreading into abdominal segment 2 (see Fig 2G). Multiphoton microscopy images were initially processed with ImSpector software (LaVision Bio Tec) to compile channels, and exported files were further processed using Imaris software (Bitplane) for 3D visualization.

Each movie was rotated and aligned along the embryonic AP axis for tracking analysis. For analysis of migration in the pre-gb and gb in the control and *Dfos*^*2*^ mutant, embryos were synchronized using the onset of germ and retraction. For vnc migration analysis, macrophages were tracked for 30 minutes from when macrophages started moving into the second abdominal segment. Only macrophages migrating along the inner edge of the vnc were analyzed.

Gb crossing time was calculated from when the macrophages align in front of the gb ectoderm in a characteristic arc, until the first macrophage had transitioned its nucleus inside the ecto–meso interphase. To see the gb edge and yolk in movies of *srpHemo-H2A*::*3xmCherry*, either *Resille*::*GFP* labeling the outlines of all cells or the autofluorescence of the yolk was used.

For analysis of gb migration in the *DfosDN* versus control macrophages, macrophages were tracked from when the first macrophage appeared between the ectoderm and the yolk sac until gb retraction started, typically 60 minutes. In the head and pre-gb, macrophage nuclei were extracted using the spot detection function, and tracks generated in 3D over time. The pre-gb and gb were defined as for macrophage counts described above. The mean position of the tracks in X- and Y- restrict analysis to each migratory zones.

Cell speed and persistence were calculated from nuclei positions using custom Python scripts as described elsewhere [106]. Briefly, instantaneous velocities from single cell trajectories were averaged to obtain a mean instantaneous velocity value over the course of measurement. The directional persistence of a trajectory was calculated as the mean cosine of an angle between subsequent instantaneous velocities:

$$I\left(v_{1},\ldots ,\;v_{l}\right)=\frac{1}{l-1}\sum_{k=1}^{l-1}cos(v_{k},v_{k+1}),$$

where *l* is the duration of the trajectory and (*v*_1_, …, *v*_*l*_) are its instantaneous velocities. Only trajectories with a minimal duration of 15 time frames were used. Calculated persistence values were averaged over all trajectories to obtain a persistence index (*I*) for the duration of measurement (with −1 being the lowest and 1 the maximum). From 3 to 6 embryos were recorded and analyzed for each genotype; numbers of control and perturbed embryos are equal in each pairwise comparison.

#### Measurement of junctional Phalloidin

The junctional intensity of F-actin (Phalloidin) was calculated using linescan analysis as previously described [107] with the following changes. The line was approximately 5 μm and was always drawn in the middle slice of the Z stack (1 μm resolution) of the macrophage–macrophage junction. For every line, a Gaussian fit was applied and maximum intensities across the cell junction were then normalized against average intensities of F-actin (Phalloidin) staining in the stereotypical gb area of approximately 50 × 50 μm^2^ in each embryo. Analyses were carried out using standard Fiji software. From 4 to 5 embryos were analyzed per genotype. Macrophages in the pre-gb or gb entry zones were analyzed.

### Measurement of F-actin reporters

To quantify cortical F-actin intensity in living embryos, a *srpHemo-moe*::*3xmCherry* reporter line [33] was crossed into a background of macrophages expressing *DfosDN*, *cher RNAi*, or *TM4SF* RNAi. Embryos were collected for 5 hours 30 minutes at 29°C, dechorionated in 50% bleach for 5 minutes, rinsed thoroughly with water, and aligned laterally side by side under a stereomicroscope using a fluorescence lamp to check for the presence of mCherry. Aligned embryos were then mounted as described in the live imaging section above. To image Moe::3xmCherry, a Zeiss LSM800 inverted microscope was used with the following settings: Plan-APOCHROMAT 40x/1.4 Oil, DIC, WD = 0.13 objective, 1.5× zoom, 1,025 × 1,025 pixel, speed 8, heating chamber set to 29°C, z-interval 1 μm. Laser settings were kept constant in all experiments. Images were acquired during macrophage invasion into the gb (St 12). Pseudo-coloring was conducted for the mCherry red channel. Each pixel in the image has a color ascribed to it via the fire “Look Up Table” translating the level of intensity of the mCherry channel into a defined amount of each color. The highest intensity of the image is represented as very bright yellow, and all other gray values are depicted as colors on the scale accordingly.

For quantification of Moe::3xmCherry intensity, an ROI was drawn in Fiji software around macrophages at the gb entry site in 20 z-stacks for each embryo. The area mean intensity was measured in all ROIs, and the average/embryo was calculated. To normalize fluorescence intensities per batch, the average intensity/embryo of all ROIs in each sample was divided by the arithmetic mean of the average intensity/embryo of all ROIs in the control per batch. The normalized average intensities/embryo were then compared to each other using a *t* test with Welch’s correction for *DfosDN* and one way-ANOVA for *cher RNAi* and *TM4SF RNAi*.

#### Quantification of membrane localization of DiaRBD::GFP and Dia::GFP

Methanol-fixed St 11 embryos were mounted either after staining with GFP antibody (Dia::GFP) or without staining (DiaRBD::GFP) and imaged with a Zeiss Inverted LSM800, Plain-Apochromat 63X/1.4 Oil Objective at an XY-resolution of 0.1 μm and a Z-resolution of 1 μm (approximately 15 μm total stack). All macrophages within 40 μm of the gb were analyzed. For the quantification of the levels of DiaRBD or the complete Dia protein at the plasma membrane versus the cytoplasm, confocal images were processed using Fiji and MATLAB-R2017b (MathWorks). Individual focal planes were used to segment a profile corresponding to an 8-pixel wide line drawn across the single outer membrane of individual macrophages chosen such that the extracellular portion of the line extended into surrounding tissue or space and not another macrophage. The corresponding intensity profiles of the Myr::Tomato and Dia::GFP or DiaRBD::GFP channels were extracted in Fiji using a custom macro and analyzed further using a custom MATLAB script. The membrane region was defined by finding the maximal value in the Tomato intensity profile and centering a 0.8-μm interval around it. The background was calculated for each GFP profile as the mean intensity in the 2 μm outside the cell, flanking the membrane region, and substracted from the entire profile. The integrated Dia::GFP or DiaRBD::GFP intensity at the membrane was calculated within the 0.8-μm interval defined above. The integrated cytoplasmic Dia::GFP or DiaRBD::GFP level was calculated as the mean intensity of 2 μm of the GFP profile inside the cell flanking the membrane region. Image analysis scripts are publicly available at https://github.com/Axmasha/Image_analysis_scripts.

#### Cell aspect ratio analysis and imaging actin dynamics

Laterally oriented embryos were used to measure the maximal length and width of macrophages expressing *UAS-CLIP*::*GFP* under the control of *srpHemo-Gal4*. Briefly, 3D-stacks with 1 μm Z resolution were acquired every 35 to 45 seconds for approximately 1 hour. As the strength of the GAL4 expression increased over time, laser power was adjusted during acquisition to reach the best possible quality of visualization. Images acquired from mutiphoton microscopy were initially processed with ImSpector software (LaVision Bio Tec) to compile channels from the imaging data.

We started measuring from the time the cell body of the first macrophage fully appeared at the interface between the ectoderm and mesoderm and yolk sac until it had moved 30 μm along the ectoderm mesoderm interface. At each time frame, a line was drawn in Fiji along the longest dimension of the macrophage in the direction of its front-rear polarization axis, denoted the maximal cell length, and along the orthologonal longest dimension, which was considered maximal cell width. We did not observe long CLIP::GFP protrusions, but when a small protrusion was present, it was not included in the length measurement; within this gb region, the front of the first macrophage was clearly outlined with CLIP::GFP. The border between the first and second entering macrophages was drawn based on the uninterrupted intense line of CLIP::GFP at the base of the first macrophage; only cells with a clearly visible border were measured. The length-to-width ratio was quantified for each time frame, and a probability density function was plotted: 5 embryos were recorded for each genotype.

#### Imaging the actin protrusion

Laterally oriented embryos expressing *srpHemo-Gal4 UAS-LifeAct*::*GFP* were used to image macrophage actin live with a 3D-stack resolution of 1 μm. See above description of CLIP::GFP labeled macrophage imaging for laser power and image compilation. Laser power was also increased further in the DfosDN samples to enhance actin visualization. We measured the length of the filopodia-like protrusion of the first entering macrophage with Imaris software (Bitplane) from the time when the protrusion was inserted into the ectoderm, mesoderm, and yolk sac interface until the macrophage started to translocate its cell body into that location.

### FACS sorting of macrophages

Adult flies of either *w; +; srpHemo-Gal4, srpHemo-3xmCherry/+* or *w; +; srpHemo-Gal4*, *srpHemo-3xmCherry/ UAS-DfosDN* genotypes were placed into plastic cages closed with apple juice plates with applied yeast to enhance egg laying. Collections were performed at 29°C for 1 hour, then kept at 29°C for additional 5 hours 15 minutes to reach stage 11 to early stage 12. Embryos were harvested for 2 days with 6 to 7 collections per day and stored meanwhile at +4°C to slow down development. Collected embryos were dissociated and the macrophages sorted as previously described [33]. About 1 to 1.5 × 10^5^ macrophages were sorted within 30 minutes.

### Sequencing of the macrophage transcriptome

Total RNA was isolated from FACS-sorted macrophages using Qiagen RNeasy Mini kit (Cat No. 74104). The quality and concentration of RNA was determined using Agilent 6000 Pico kit (Cat No. 5067–1513) on an Agilent 2100 Bioanalyzer: on average about 100 ng of total RNA was extracted from 1.5 × 10^5^ macrophages. RNA sequencing was performed by the CSF facility of Vienna Biocenter according to standard procedures (https://www.vbcf.ac.at/facilities/next-generation-sequencing/) on 3 replicates. Briefly, the cDNA library was synthesized using QuantSeq 3′ mRNA-seq Library Prep kit and sequenced on the Illumina HiSeq 2500 platform. The reads were mapped to the *Drosophila melanogaster* Ensembl BDGP6 reference genome with STAR (version 2.5.1b). The read counts for each gene were detected using HTSeq (version 0.5.4p3). Flybase annotation (r6.19) was used in both mapping and read counting. Counts were normalized to arbitrary units using the TMM normalization from edgeR package in R. Prior to statistical testing, the data were voom transformed, and then the differential expression between the sample groups was calculated with limma package in R. The functional analyses were done using the topGO and gage packages in R [108,109]. RNA sequencing data have been deposited at GEO as GSE182470.

### qRT-PCR analysis of mRNA levels in murine bones and osteosarcomas

RNA isolation and qPCR was performed from bones of wild-type C57BL/6 mice and from bones and OS of H2-c-fosLTR as previously described with the primers in Table 1 [110].

**Table 1:** Primers used for qPCR of mouse bones and osteosarcomas.

Primer	Sequence
Fos fw	ATGGTGAAGACCGTGTCAGG
Fos_rv	GTTGATCTGTCTCCGCTTGGA
Flna_fw	GTCACAGTGTCAATCGGAGGT
Flna_rv	TTGCCTGCTGCTTTTGTGTC
Flnb_fw	TTCTACACTGCTGCCAAGCC
Flnb_rv	CTGTAACCCAGGGCCTGAATC
Flnc_fw	CATCACCCGGAGTCCTTTCC
Flnc_rv	CTCTGTGCCCTTTGGACCTT
Tspan6_fw	TCGAACTAGTTGCCGCCATT
Tspan6_rv	CCGCAACAATGCAACGTACT
Gstt3_fw	GGAGCTCTACCTGGACCTGA
Gstt3_rv	AAGATGGCCACACTCTCTGC
Eva1c_fw	GTTGCCTACGCATGTGTTCC
Eva1c_rv	CCGATGCAGACACTGGACAT
Tspo_fw	GTATTCAGCCATGGGGTATGG
Tspo_rv	AAGCAGAAGATCGGCCAAGG
Tbp_fw	GGGGAGCTGTGATGTGAAGT
Tbp_rv	CCAGGAAATAATTCTGGCTCAT

### Statistics and repeatability

Mouse experiments: Data are shown as mean ± SEM. One-way ANOVA followed by Tukey multiple comparisons posttest was applied to compare experimental groups. Statistical analysis was performed using GraphPad Prism 6.0 software. A *p*-value <0.05 was considered statistically significant (*p < 0.05, **p < 0.01, ***p < 0.001, ****p < 0.0001).

*Drosophila* experiments: Statistical tests as well as the number of embryos, cells, tracks, or contacts assessed are listed in the figure legends. All statistical analyses were performed using GraphPad PRISM or R Studio, and significance was determined using a 95% confidence interval. No statistical method was used to predetermine sample size.

Representative images of Dfos antibody staining were analyzed per replicate per genotype and in situ hybridization are from experiments that were repeated 2 times with many embryos with reproducible results. *Dfos* mutant analyses in Fig 1 and S1 Fig are from experiments that were repeated 2 to 3 times. In live imaging experiments in Fig 2 and S2 Fig, 3 to 7 embryos for each genotype were analyzed; each embryo was recorded on a separate day. FACS sorting of macrophages from embryos was conducted in 3 replicates, from which RNA samples were prepared for RNA sequencing. Experiments in Fig 4 and S4 Fig were repeated at least 3 times, with representative images and plots of phalloidin immunostaining from experiments that were repeated 4 times. In the LifeAct::GFP protrusion live imaging experiment in Fig 5 and S5 Fig, 3 to 5 embryos were analyzed for each genotype. In CLIP::GFP live imaging experiments in Fig 5 and S5 Fig, 5 to 6 embryos were analyzed for each genotype for the cell aspect ratio in the gb zone, and 2 embryos in the pre-gb zone and for tracking of the front versus rear speed. Each embryo was recorded on a separate day. The Lamin overexpression in S5 Fig and the Lamin knockdown rescue experiments in Fig 5G were repeated at least 3 times. The gb rescue experiment in Fig 5H was repeated at least 4 times.

## Supporting information

S1 Table Macrophages acquire distinct TF profiles at different stages of embryonic development. Comparison of TF mRNA expression in macrophages at stages 11–12 and 13–16, based on data in [21]. TFs expressed in the macrophages only at stages 11–12 are highlighted in green. TFs expressed in macrophages only at stages 13–16 are highlighted in blue. Function is annotated only for TFs expressed in macrophages at stages 11–12. TF, transcription factor.
(TIF)

S2 Table Genes up-regulated in macrophages expressing DfosDN. Genes are ordered according to the adjusted p-value from the RNA sequencing. Function is based on Flybase assignments [23]. The murine ortholog with the top score in UniProt BLAST is shown in the rightmost column.
(TIF)

S1 Raw images Raw images of western blots shown in S4A Fig. Three original uncropped western blots of St 11 embryo extracts from *srpHemo-moe*::*3xmCherry* expressing either CD8::GFP (ctrl) or DfosDN in macrophages. Rightmost western blot also contains a w- lane. Top row shows blots probed with an mCherry antibody, bottom row the same blots probed with a profilin antibody as a loading control. Cropped versions of the blots are shown in S4A Fig.
(PDF)

S1 Data Data used to plot all graphs and to perform statistical analyses. This Excel file contains the raw data of the quantification of embryo macrophage counts and linescan analyses along with movie outputs. Each tab in the file names the figure panel whose graph is based on the data shown in that chart.
(XLSX)

S2 Data RNA sequencing data. Compendium of the RNA sequencing data obtained from FACSed macrophages from Stages 11–12 *srpHemo-3xmCherry* control embryos and those expressing DfosDN in macrophages. The mean from 3 samples is shown for each genotype, organized by Flybase IDs (Fbgn), along with statistical analyses.
(XLSB)

S1 Fig Dfos does not affect the total number of macrophages, or their number in the pre-gb zone and along the vnc. (A) Dfos protein (green) is detected with an antibody in macrophages (magenta) in embryos from the stages as indicated. (B-I) Quantification in mid St 12 embryos. (B) The number of macrophages (green) in the pre-gb zone (outlined by a black dotted line in the schematic on the left) showed no significant change in *Dfos^2^* mutant embryos compared to the control (p = 0.37) SD: 6, 7. (C) The total number of macrophages (see schematic at left) was not altered from that in the control embryos expressing DfosDN in macrophages (p = 0.12). SD: 60, 120. (D, E) The number of macrophages (green) along the vnc (outlined by black dotted line in the schematic on the left) shows no significant difference between the control and (D) macrophages that express DfosDN or (E) either of 2 RNAi lines against Dfos. (D) *DfosDN* p = 0.88, 0.99, >0.99. *Dfos RNAi^1^* (TRiP HMS00254) p = 0.21, 0.06, 0.11, 0.072, 0.033, 0.30, 0.56. *Dfos RNAi^2^* (TRiP JF02804) p = 0.34, 0.15, 0.83, 0.27, 0.47, 1.0, 0.45. (D) SD: Ctrl 3, 3, 3, 0.8; *DfosDN* 6, 3, 0.7. (E) SD: Ctrl 6, 3, 3, 3, 2, 0.3; *Dfos RNAi^1^* 6, 3, 3, 3, 2, 2, 0.3; *Dfos RNAi^2^* 6, 2, 3, 2, 3, 1, 0.4. (F, G) Macrophage numbers in the pre-gb (see schematic at left) are increased compared to the control for lines expressing (F) DfosDN or (G) one of 2 different *UAS-Dfos RNAi* constructs in macrophages under *srpHemo-GAL4* control. (F) p = 0.04, SD: 19, 29. (G) *Dfos RNAi^1^* p < 0.0009, *Dfos RNAi^2^* p < 0.0001. SD: 12, 9, 14. (H) Macrophage numbers in the gb are not significantly altered compared to the control upon overexpression of Dfos in macrophages (p = 0.14). SD: 22, 14. (I) Macrophage numbers in the gb for lines expressing one of 2 different *UAS-Dfos RNAi* constructs in macrophages under *srpHemo-GAL4* control and lines, which additionally express *UAS-GFP*. Control vs. *mac>Dfos RNAi^1^* (TRiP HMS00254) or Control vs. *mac>Dfos RNAi^2^* (TRiP JF02804), p < 0.0001. *mac>Dfos RNAi^1^* vs. *mac>Dfos RNAi^1^ + GFP* or *mac>Dfos RNAi^2^* vs. *mac>Dfos RNAi^2^ + GFP*, p > 0.99. SD: 33, 47, 34. The effect of each Dfos RNAi was eliminated upon simultaneous expression of another UAS construct. Macrophages are labeled using either *srpHemo-Gal4* driving *UAS-GFP* or *srpHemo-H2A*::*3xmCherry*. “mac>” indicates *srpHemo-GAL4* driver expressing UAS constructs specifically in macrophages. Histograms show mean ± SEM ***p < 0.005, **p < 0.01, *p < 0.05. Unpaired t test was used for statistics, except for G, I, which used one-way ANOVA. The number of embryos analyzed for that genotype is shown within each column in the graphs. In D, n = 6 embryos for the control and n = 9 for Dfos DN. In E, n = 9 embryos for control, 15 and 11 for Dfos RNAis. Scale bar in A: 10 μm. The data underlying the graphs can be found in S1 Data. ctrl, control; gb, germband; ns, not significant; RNAi, RNA interference; SD, standard deviation; SEM, standard error of the mean; vnc, ventral nerve cord.
(TIF)

S2 Fig Dfos facilitates macrophage motility during initial invasion into the tissue. (A) Quantification reveals that the directional persistence of macrophages expressing DfosDN (0.58) is unchanged (0.56) in the pre-gb area (p = 0.66) but decreased during gb entry (0.65) (0.72), p = 0.038 and along the vnc (0.54) compared to the control (0.61), p = 0.00026. Left schematic shows pre-gb area in yellow, gb entry outlined in solid line. Boxed area in right schematic shows analyzed area of vnc. (B) Movie stills showing wild-type and *Dfos^2^* macrophages entering the gb (outlined by the dashed line). Time in minutes shown in the top right corner of each image. (C) Quantification of macrophage speed shows a significant reduction in the speed of *Dfos^2^* macrophages in the pre-gb zone and at gb entry, but none in the head. Regions analyzed indicated in left schematic. Speed in head: control = 2.59 μm/min, *Dfos^2^* = 2.68 μm/min, p = 0.40; speed in pre-gb = 3.38 μm/min, *Dfos^2^* = 2.47 μm/min, p = 2.38e-06; speed in gb entry: control = 2.35 μm/min, *Dfos^2^* = 1.62 μm/min, p = 0.0003. Macrophages are labeled using *srpHemo-H2A*::*3xmCherry*. Histograms show mean ± SEM. ****p < 0.0001, ***p < 0.005, **p < 0.01, *p < 0.05. Unpaired t test was used for statistics. The number of analyzed macrophages for each genotype shown within each graph column. Tracks were obtained from movies of 3 embryos each for control and *mac>DfosDN* for pre-gb entry in A, 4 each for gb entry in A, 3 each for the vnc in A, 4 each of control and 4 *Dfos^2^* embryos for head and pre-gb in C, and 3 embryos each for gb entry in C. Scale bars: 10 μm. The data underlying the graphs can be found in S1 Data. ctrl, control; gb, germband; ns, not significant; SEM, standard error of the mean; vnc, ventral nerve cord.
(TIF)

S3 Fig Dfos regulates macrophage gb invasion through actin cytoskeleton-associated proteins. (A-C) Comparative mRNA expression levels as determined from RNA sequencing analysis of FACS-sorted wild-type macrophages and those expressing DfosDN, n = 3 biological replicates. (A, B) Genes down-regulated in macrophages expressing DfosDN are shown, separated into those with (A) strong and (B) moderate expression in wild-type macrophages. (C) Expression levels of Drosophila formin family genes are unchanged. Fold enrichment is normalized. p-values: *Dhc36C* 0.02, *CG14204* 0.03, *CG42402* 0.04, *CR43767* 0.046, *TM4SF* 0.03, *CG42260* 0.0011, *cher* 0.046, *GstT4* 0.018, *Xrp1* 0.0011, *Tspo* 0.046, *CG31337* 0.046. *Frl, DAAM, dia, capu* all >0.99. (D, E) Quantification of the macrophage numbers in (D) the pre-gb zone and (E) along the vnc from embryos expressing RNAi against *cher* (KK 107451), or *TM4SF* in macrophages (KK 102206) driven by *srpHemo-Gal4* shows no significant alteration. The number in the column in (D) corresponds to the number of embryos analyzed. Control vs. *cher RNAi* p = 0.33. Control vs. *TM4SF RNAi* p = 0.05. Control vs. *cher/TM4SF RNAi* p = 0.67. (D) SD: 20, 20, 19, 13. For (E), n = 13 embryos for control and n = 15 for each *cher RNAi* and *TM4SF RNAi*. Control vs. *cher RNAi* p = 0.97 for T1, p = 0.33 for T2, p = 0.88 for T3. Control vs. *TM4SF RNAi* p = 0.52 for T1, p = 0.76 for T2, p = 0.35 for T3. SD: ctrl 6.5, 5.4, 0.6; *cher RNAi* 5.0, 3.3, 0.8; *TM4SF RNAi* 4.4, 4.9, 1.9. (F-I) q-PCR analysis of mRNA extracted from the bones of mice that are wild type, tg for MHC c-fos, viral 3′ UTR, and those in which c-fos transgenesis has led to an OS. Analysis of mRNA expression shows that (F) higher Fos levels in OS correlate with higher levels of (G) the glutathione S transferase Gstt3, and (H) the slit receptor Eva1c, but not (I) Tspo. Bone and OS RNA isolated from the same transgenic mouse, n = 4 mice per group, age 5 to 6 months. p-values = 0.86, 0.0028, 0.0013 in (F), 0.79, 0.0001, 0.0003 in (G), 1.0, 0.054, 0.049 in (H), 0.37, 0.33, 0.040 in (I). SD: 0.7, 0.6, 2.6 in (F); 0.2, 0.3, 1.1 in (G); 0.4, 0.2, 1.5 in (H); 0.1, 0.2, 0.2 in (I). Histograms show mean ± SEM ***p < 0.005, **p < 0.01, *p < 0.05. Unpaired t test or one-way ANOVA with Tukey post hoc were used for statistics of quantifications. Significance is based on adjusted p-values. The data underlying the graphs can be found in S1 Data. ctrl, control; gb, germband; ns, not significant; OS, osteosarcoma; RNAi, RNA interference; SD, standard deviation; SEM, standard error of the mean; tg, transgenic; vnc, ventral nerve cord; wt, wild-type.
(TIF)

S4 Fig Dia does not affect macrophage numbers in the pre-gb zone and along the vnc. (A) Three western blots probed with an mCherry antibody of St 11 embryo extracts from *srpHemo-moe*::*3xmCherry* expressing either CD8::GFP (ctrl) or DfosDN in macrophages. Left western blot also contains w- lane. Original uncropped western blots can be found in S1 Raw images. (A’) Quantitation of the western blots. We observed no significant change in the expression of the Moe protein reporter when Dfos function is inhibited. (B) Expressing Dia-CA in macrophages in *Dfos^1^* or *Dfos^2^* embryos completely rescued the macrophage gb invasion defect. p-values: Control vs. *Dfos^1^* or vs. *Dfos^2^* p = 0.0004 or p = 0.0055, respectively; Control vs. *Dfos^1^ mac>DiaCA* or vs. *Dfos^2^ mac>DiaCA* p > 0.999; *Dfos^1^* vs. *Dfos^1^ mac>DiaCA* p = 0.0005; *Dfos^2^* vs. *Dfos^2^ mac>DiaCA* p = 0.035. SD: 20, 23, 18, 19, 7.8. (C, D) There was no significant change in the number of macrophages in (C) the pre-gb zone or (D) along the vnc in embryos expressing either of 2 different RNAi lines against dia expressed in macrophages. Pre-gb: Control vs. *dia RNAi^1^* p = 0.54, Control vs. *dia RNAi^2^* p = 0.77. vnc: Control vs. *dia RNAi^1^* p = 0.99, Control vs. *dia RNAi^2^* p = 0.95. *RNAi*^1^ = TRiP HMS05027, *RNAi*^2^ = TRiP HMS00308. (C) SD: 9, 12, 13. (D) SD: Ctrl 5.2, 6.4, 2.5, 0.4; *dia RNAi^1^* 5.6, 6.8, 1.7, 0.2; *dia RNAi^2^* 5.1, 4.9, 2.1, 0.6. (E, F) Two further examples of line profiles used for the determination of the membrane-to-cytoplasmic ratios in Fig 4N and 4P. Line intensity profiles of (E) Dia::GFP or (F) DiaRBD::GFP (green) and membrane myr::Tomato (magenta) across the edge of macrophages expressing either lacZ (Control), RhoDN, DfosDN, *cher RNAi*, or *TM4SF RNAi* as shown in the schematic in E. Line length approximately 8 μm. Blue lines indicate mean GFP intensity on the membrane and in cytoplasm. Histograms show mean ± SEM ***p < 0.005, **p < 0.01, *p <0.05. One-way ANOVA with Tukey post hoc was used for statistics of quantification. The number in each column corresponds to the number of analyzed embryos. “mac>” indicates *srpHemo-GAL4* driver expressing UAS constructs in macrophages. Macrophages are labeled using *srpHemo-H2A*::*3xmCherry*. The data underlying the graphs can be found in S1 Data. ctrl, control; gb, germband; ns, not significant; RNAi, RNA interference; SD, standard deviation; SEM, standard error of the mean; vnc, ventral nerve cord.
(TIF)

S5 Fig Dfos controls cell shape in macrophages. (A) Representative image showing actin protrusions of the first macrophage entering the gb in the control and in lines expressing DfosDN in macrophages. Actin was visualized by *srpHemo-Gal4* (“*mac>*”) driving *UAS-LifeActGFP*. White stars indicate the tip of each actin protrusion. Scale bar 5 μm. (B) Microtubules are labeled with *srpHemo-Gal4* driving *UAS-CLIP*::*GFP*. Spatially matched stills of the first macrophage expressing DfosDN and control extending protrusions into the gb slightly before entering with the body of the cell. As DfosDN macrophages have a delay in entry, the stills from the DfosDN movie are from a later developmental time point than the control. (C) Quantification of macrophage maximum length and maximum width shows that DfosDN expressing macrophages are 23% longer and 12% thinner than wild-type macrophages inside the gb (indicated in schematic above by dashed box). Control vs. DfosDN maximum length p = 0.0005, SD: 3.4, 5.7; control vs. DfosDN maximum width p = 0.0025, SD: 1.3, 1.0. (D) Quantification of the maximum length and maximum width of macrophages in the pre-gb zone (indicated in schematic by dashed box) shows that macrophages expressing DfosDN are 9% shorter and 9% thinner than wild-type macrophages. Control vs. DfosDN maximum length p = 0.0095, SD: 2.2, 2.0; control vs. DfosDN maximum width p = 0.005, SD: 2.3, 1.9. (E) Overexpression of *UAS-Lam* in macrophages through *srpHemo-Gal4* (*mac>*) causes no change in their number in the gb compared to the control. p = 0.65, SD: 15, 18. Histograms show mean ± SEM ***p < 0.005, **p < 0.01, *p < 0.05. Unpaired t test was used for statistics of quantification. The number of measurements per genotype is shown in each columns. The data underlying the graphs can be found in S1 Data. ctrl, control; gb, germband; ns, not significant; SD, standard deviation; SEM, standard error of the mean.
(TIF)

S6 Fig Model of protein interactions at the macrophage cortex. Proposed interactions of proteins at the cell cortex in wild-type macrophages during gb infiltration as shown in Fig 6. Direct binding between 2 proteins is indicated by a line, signaling between the interaction partners is represented as an arrow. These interactions and the resulting model in Fig 6 are based on the papers at the end of this legend next to the corresponding number shown for each linkage. The Tetraspanin TM4SF can cluster adhesion receptors such as Integrins at the membrane and lead to the recruitment and activation of Rho GTPases. Rho GTPases can bind and activate the formin Dia leading to F-actin polymerization. In addition, Integrin can bind filamins (Cher), which can bind to and thereby recruit RhoGEF to the membrane. Rho GEFs can in turn bind to and activate Rho GTPases. References for listed interactions: 1, Tetraspanins-Integrin) [71,76]. 2, Tetraspanins-Rho GTPases) [72–74]. 3, Tetraspanins-Filamins) [49,50]. 4, Integrin-Filamins) [35,68]. 5, Rho1 GTPase-Dia in Drosophila) [45,81]. 6 and 8, Rho GEF-Rho GTPases) [83]. 7, Formins-Filamins) [85,86]. 9, Filamins-RhoGEFs) [47,48]. Cher, Cheerio; gb, germband.
(TIF)

S1 Movie Dfos facilitates macrophage motility during initial invasion into the gb tissue. Movies corresponding to stills shown in Fig 2A. Macrophages (green) labeled using *srpHemo-H2A*::*3xmCherry* are imaged while entering the gb in control embryos (left) and embryos in which macrophages express DfosDN (right). Time in minutes is indicated in the upper right corner. Scale bar: 10 μm. DfosDN, dominant negative version of Dfos; DN, dominant negative; gb, germband.
(AVI)

S2 Movie Dfos does not affect macrophage migration along the vnc. Movies corresponding to stills shown in Fig 2G. Macrophages (green) labeled by *srpHemo-Gal4* driving *UAS-GFP*::*nls* are imaged during their migration along the segments of the vnc in control embryos (left) and embryos in which DfosDN is expressed in macrophages (right). Time in minutes is indicated in the upper right corner. Scale bar: 10 μm. DfosDN, dominant negative version of Dfos; vnc, ventral nerve cord.
(AVI)

S3 Movie Macrophages in *Dfos^2^* mutants invade gb more slowly. Movies corresponding to stills shown in S2B Fig. Macrophages (green) labeled by *srpHemo-Gal4* driving *UAS-GFP*::*nls* are imaged while entering the gb in control embryos (left) and *Dfos^2^* mutant embryos (right). Time is indicated in minutes. Scale bar: 10 μm. gb, germband.
(AVI)

S4 Movie DfosDN expressing macrophages make long actin protrusions during gb entry. Movies corresponding to stills shown in S5A Fig. F-actin in macrophages (green) labeled with *srpHemo-Gal4* driving *UAS-LifeAct*::*GFP* is imaged during gb entry in control embryos (left) and embryos with macrophages expressing DfosDN (right). Note the extended protrusion of the DfosDN expressing macrophages. Time is indicated in minutes. Scale bar: 10 μm. DfosDN, dominant negative version of Dfos; gb, germband.
(AVI)

S5 Movie Dfos controls cell shape in macrophages. Movies corresponding to stills shown in Fig 5B and 5C and S5B Fig. Microtubules of macrophages are labeled with *srpHemo-Gal4* driving *UAS-CLIP*::*GFP*. They are imaged during gb entry in control embryos (left) and embryos with macrophages expressing DfosDN (right). Note the extended shape of the DfosDN expressing macrophages. Time is indicated in minutes. Scale bar: 10 μm. DfosDN, dominant negative version of Dfos; gb, germband.
(AVI)

## Abbreviations

bZIP: basic leucine zipper domain
CA: constitutively active; Dia, Diaphanous
Cher: Cheerio
DN: dominant negative
ECM: extracellular matrix
FA: focal adhesion
gb: germband
mac: macrophage
MMP: matrix metalloproteinase
OS: osteosarcoma
RBD: Rho Binding Domain
RNAi: RNA interference
vnc: ventral nerve cord
