# Correction: Fos regulates macrophage infiltration against surrounding tissue resistance by a cortical actin-based mechanism in *Drosophila*

**DOI:** 10.1371/journal.pbio.3001818

**Published:** 2022-09-21

**Authors:** The PLOS Biology Staff

## Notice of republication

This article was republished on February 7, 2022, to correct an error in the author list footnotes. The publisher apologizes for the errors. Please download this article again to view the correct version.

## References

[1] Belyaeva V, Wachner S, Gyoergy A, Emtenani S, Gridchyn I, Akhmanova M, et al. (2022) Fos regulates macrophage infiltration against surrounding tissue resistance by a cortical actin-based mechanism in *Drosophila*. PLoS Biol 20(1): e3001494. 10.1371/journal.pbio.3001494 34990456 PMC8735623

