# Supplementary figures and images for: Fos regulates macrophage infiltration against surrounding tissue resistance by a cortical actin-based mechanism in Drosophila
Source: PLoS Biol. 2022 Jan 6;20(1):e3001494. doi: 10.1371/journal.pbio.3001494 (PMC8735623; doi:10.1371/journal.pbio.3001494)

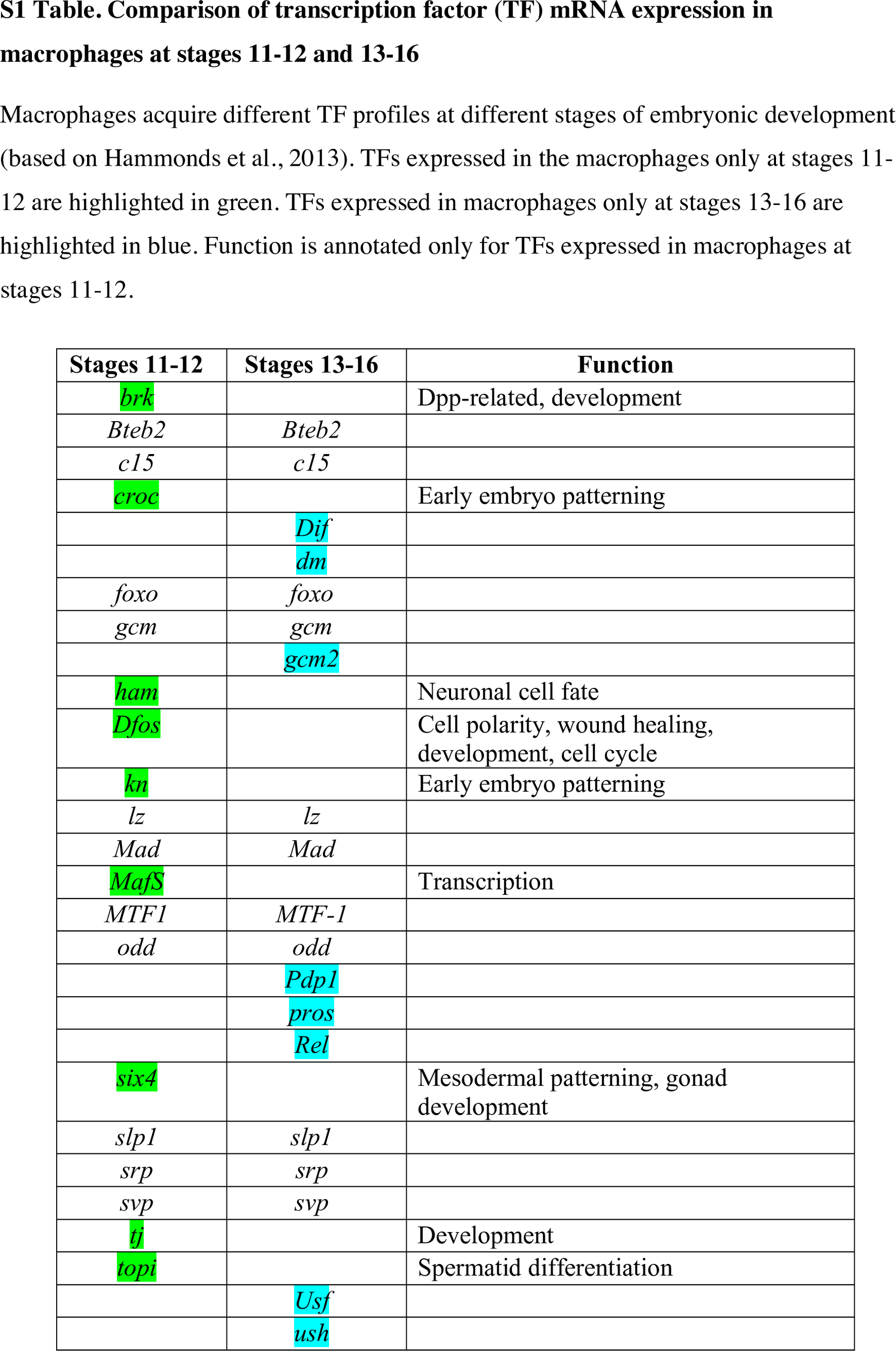

Supplement: S1 Table — Comparison of TF mRNA expression in macrophages at stages 11–12 and 13–16, based on data in [21]. TFs expressed in the macrophages only at stages 11–12 are highlighted in green. TFs expressed in macrophages only at stages 13–16 are highlighted in blue. Function is annotated only for TFs expressed in macrophages at stages 11–12. TF, transcription factor. (TIF) [file pbio.3001494.s001.tif]

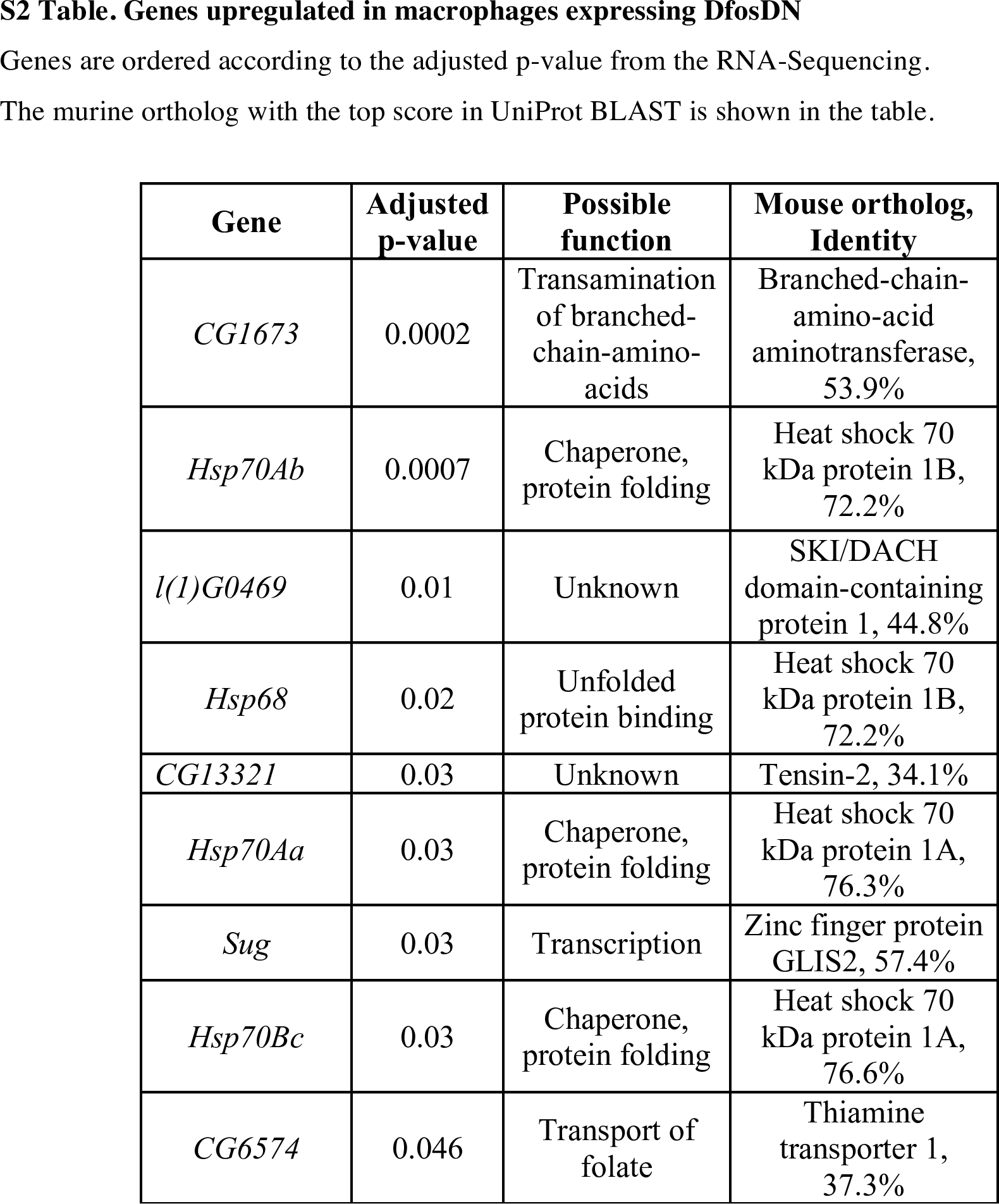

Supplement: S2 Table — Genes are ordered according to the adjusted p-value from the RNA sequencing. Function is based on Flybase assignments [23]. The murine ortholog with the top score in UniProt BLAST is shown in the rightmost column. (TIF) [file pbio.3001494.s002.tif]

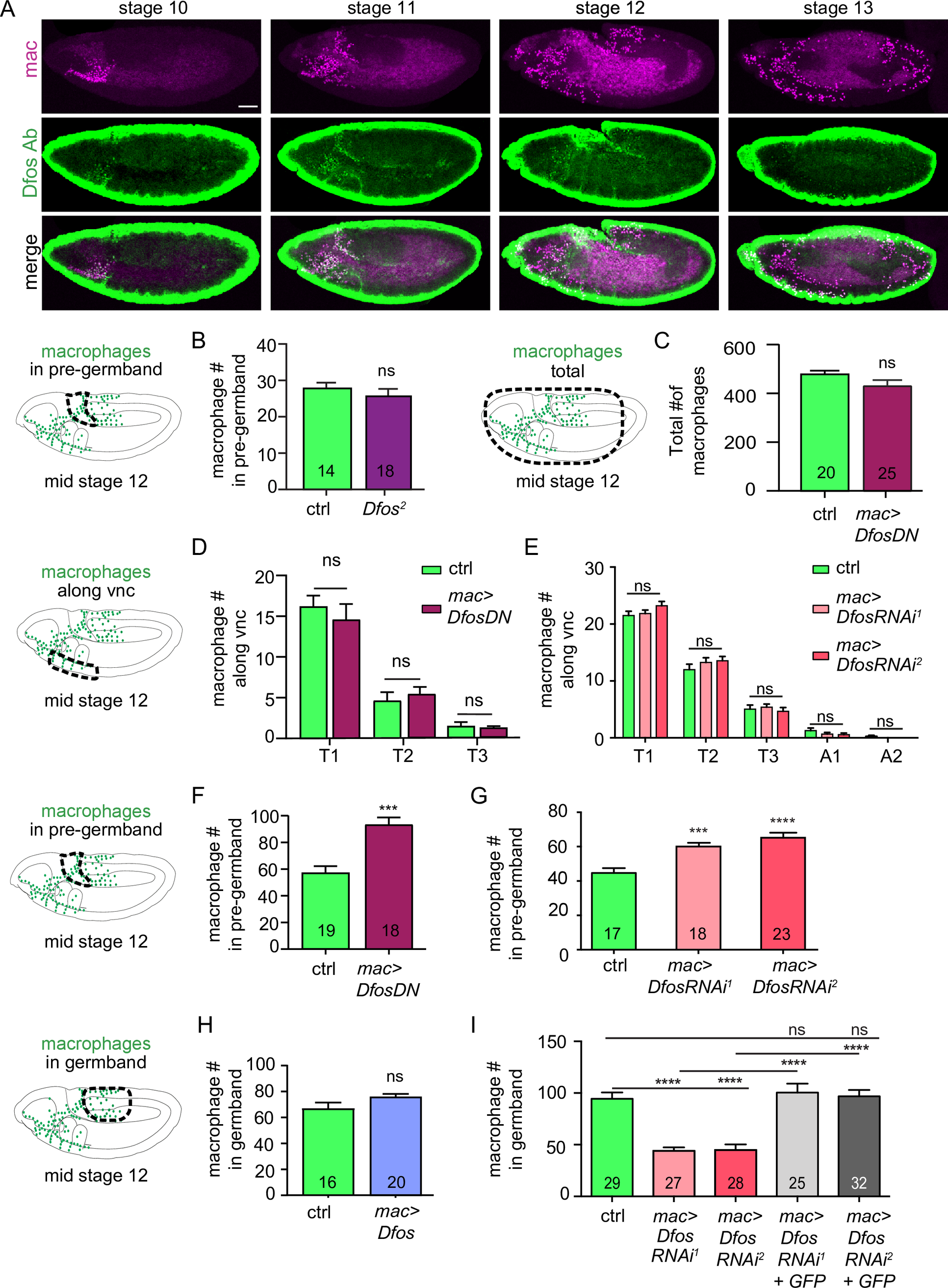

Supplement: S1 Fig — (A) Dfos protein (green) is detected with an antibody in macrophages (magenta) in embryos from the stages as indicated. (B-I) Quantification in mid St 12 embryos. (B) The number of macrophages (green) in the pre-gb zone (outlined by a black dotted line in the schematic on the left) showed no significant change in Dfos2 mutant embryos compared to the control (p = 0.37) SD: 6, 7. (C) The total number of macrophages (see schematic at left) was not altered from that in the control embryos expressing DfosDN in macrophages (p = 0.12). SD: 60, 120. (D, E) The number of macrophages (green) along the vnc (outlined by black dotted line in the schematic on the left) shows no significant difference between the control and (D) macrophages that express DfosDN or (E) either of 2 RNAi lines against Dfos. (D) DfosDN p = 0.88, 0.99, >0.99. Dfos RNAi1 (TRiP HMS00254) p = 0.21, 0.06, 0.11, 0.072, 0.033, 0.30, 0.56. Dfos RNAi2 (TRiP JF02804) p = 0.34, 0.15, 0.83, 0.27, 0.47, 1.0, 0.45. (D) SD: Ctrl 3, 3, 3, 0.8; DfosDN 6, 3, 0.7. (E) SD: Ctrl 6, 3, 3, 3, 2, 0.3; Dfos RNAi1 6, 3, 3, 3, 2, 2, 0.3; Dfos RNAi2 6, 2, 3, 2, 3, 1, 0.4. (F, G) Macrophage numbers in the pre-gb (see schematic at left) are increased compared to the control for lines expressing (F) DfosDN or (G) one of 2 different UAS-Dfos RNAi constructs in macrophages under srpHemo-GAL4 control. (F) p = 0.04, SD: 19, 29. (G) Dfos RNAi1 p < 0.0009, Dfos RNAi2 p < 0.0001. SD: 12, 9, 14. (H) Macrophage numbers in the gb are not significantly altered compared to the control upon overexpression of Dfos in macrophages (p = 0.14). SD: 22, 14. (I) Macrophage numbers in the gb for lines expressing one of 2 different UAS-Dfos RNAi constructs in macrophages under srpHemo-GAL4 control and lines, which additionally express UAS-GFP. Control vs. mac>Dfos RNAi1 (TRiP HMS00254) or Control vs. mac>Dfos RNAi2 (TRiP JF02804), p < 0.0001. mac>Dfos RNAi1 vs. mac>Dfos RNAi1 + GFP or mac>Dfos RNAi2 vs. mac>Dfos RNAi2 + GFP, p > 0.99. SD: 33, 47, 34. The [file pbio.3001494.s006.tif]

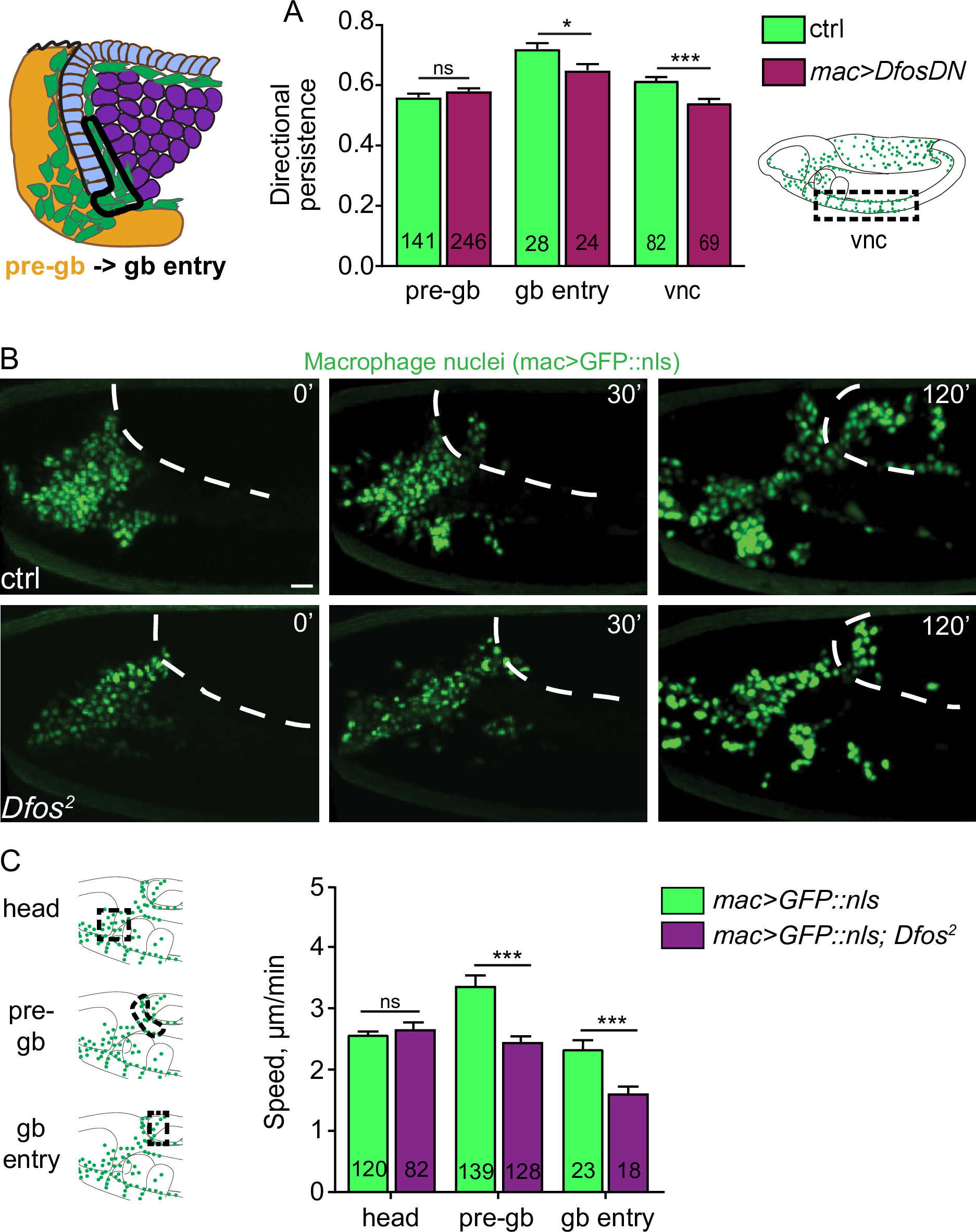

Supplement: S2 Fig — (A) Quantification reveals that the directional persistence of macrophages expressing DfosDN (0.58) is unchanged (0.56) in the pre-gb area (p = 0.66) but decreased during gb entry (0.65) (0.72), p = 0.038 and along the vnc (0.54) compared to the control (0.61), p = 0.00026. Left schematic shows pre-gb area in yellow, gb entry outlined in solid line. Boxed area in right schematic shows analyzed area of vnc. (B) Movie stills showing wild-type and Dfos2 macrophages entering the gb (outlined by the dashed line). Time in minutes shown in the top right corner of each image. (C) Quantification of macrophage speed shows a significant reduction in the speed of Dfos2 macrophages in the pre-gb zone and at gb entry, but none in the head. Regions analyzed indicated in left schematic. Speed in head: control = 2.59 μm/min, Dfos2 = 2.68 μm/min, p = 0.40; speed in pre-gb = 3.38 μm/min, Dfos2 = 2.47 μm/min, p = 2.38e-06; speed in gb entry: control = 2.35 μm/min, Dfos2 = 1.62 μm/min, p = 0.0003. Macrophages are labeled using srpHemo-H2A::3xmCherry. Histograms show mean ± SEM. ****p < 0.0001, ***p < 0.005, **p < 0.01, *p < 0.05. Unpaired t test was used for statistics. The number of analyzed macrophages for each genotype shown within each graph column. Tracks were obtained from movies of 3 embryos each for control and mac>DfosDN for pre-gb entry in A, 4 each for gb entry in A, 3 each for the vnc in A, 4 each of control and 4 Dfos2 embryos for head and pre-gb in C, and 3 embryos each for gb entry in C. Scale bars: 10 μm. The data underlying the graphs can be found in S1 Data. ctrl, control; gb, germband; ns, not significant; SEM, standard error of the mean; vnc, ventral nerve cord. (TIF) [file pbio.3001494.s007.tif]

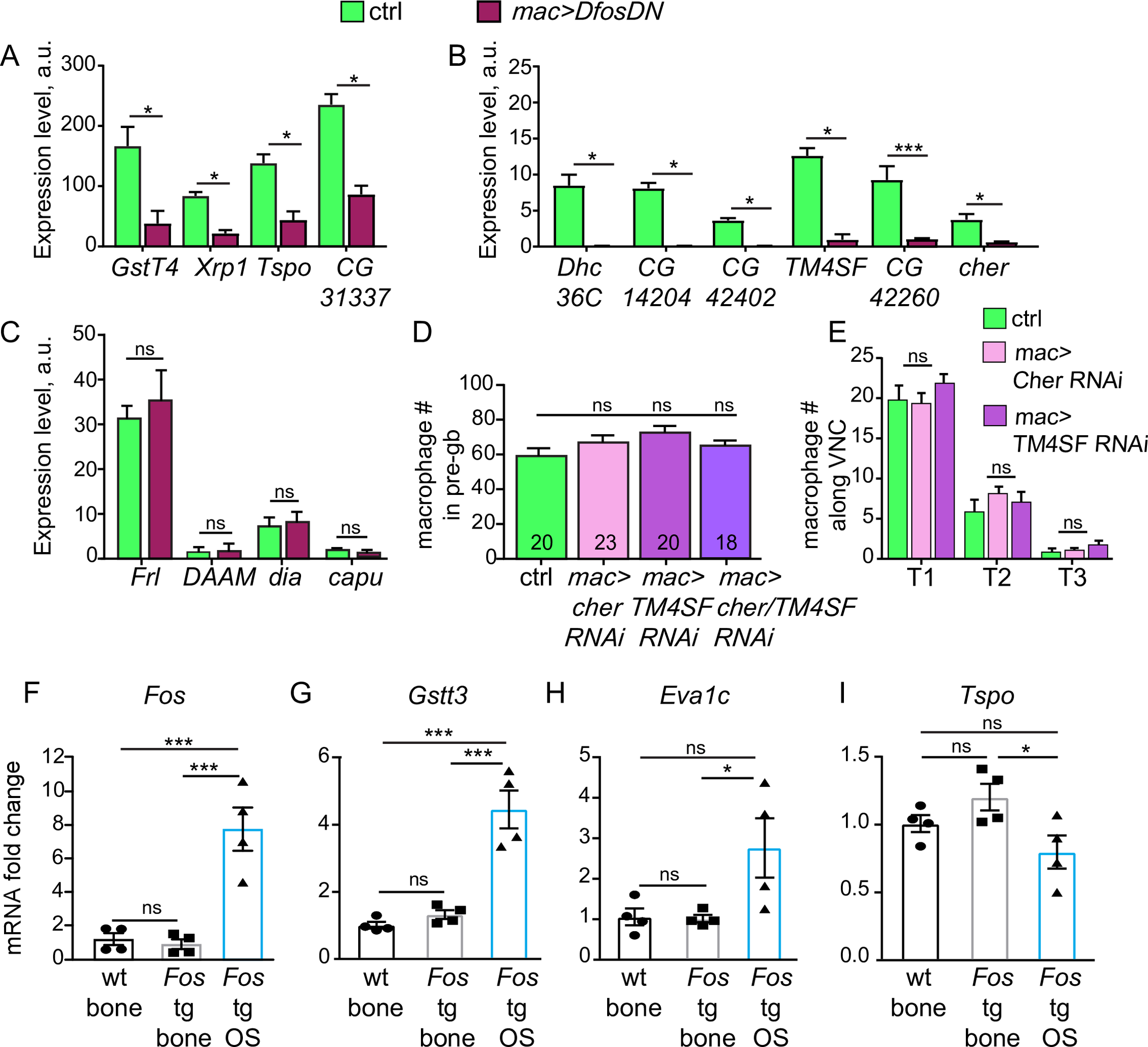

Supplement: S3 Fig — (A-C) Comparative mRNA expression levels as determined from RNA sequencing analysis of FACS-sorted wild-type macrophages and those expressing DfosDN, n = 3 biological replicates. (A, B) Genes down-regulated in macrophages expressing DfosDN are shown, separated into those with (A) strong and (B) moderate expression in wild-type macrophages. (C) Expression levels of Drosophila formin family genes are unchanged. Fold enrichment is normalized. p-values: Dhc36C 0.02, CG14204 0.03, CG42402 0.04, CR43767 0.046, TM4SF 0.03, CG42260 0.0011, cher 0.046, GstT4 0.018, Xrp1 0.0011, Tspo 0.046, CG31337 0.046. Frl, DAAM, dia, capu all >0.99. (D, E) Quantification of the macrophage numbers in (D) the pre-gb zone and (E) along the vnc from embryos expressing RNAi against cher (KK 107451), or TM4SF in macrophages (KK 102206) driven by srpHemo-Gal4 shows no significant alteration. The number in the column in (D) corresponds to the number of embryos analyzed. Control vs. cher RNAi p = 0.33. Control vs. TM4SF RNAi p = 0.05. Control vs. cher/TM4SF RNAi p = 0.67. (D) SD: 20, 20, 19, 13. For (E), n = 13 embryos for control and n = 15 for each cher RNAi and TM4SF RNAi. Control vs. cher RNAi p = 0.97 for T1, p = 0.33 for T2, p = 0.88 for T3. Control vs. TM4SF RNAi p = 0.52 for T1, p = 0.76 for T2, p = 0.35 for T3. SD: ctrl 6.5, 5.4, 0.6; cher RNAi 5.0, 3.3, 0.8; TM4SF RNAi 4.4, 4.9, 1.9. (F-I) q-PCR analysis of mRNA extracted from the bones of mice that are wild type, tg for MHC c-fos, viral 3′ UTR, and those in which c-fos transgenesis has led to an OS. Analysis of mRNA expression shows that (F) higher Fos levels in OS correlate with higher levels of (G) the glutathione S transferase Gstt3, and (H) the slit receptor Eva1c, but not (I) Tspo. Bone and OS RNA isolated from the same transgenic mouse, n = 4 mice per group, age 5 to 6 months. p-values = 0.86, 0.0028, 0.0013 in (F), 0.79, 0.0001, 0.0003 in (G), 1.0, 0.054, 0.049 in (H), 0.37, 0.33, 0.040 in (I). SD: 0.7, 0.6, 2.6 in (F); 0.2, 0.3, [file pbio.3001494.s008.tif]

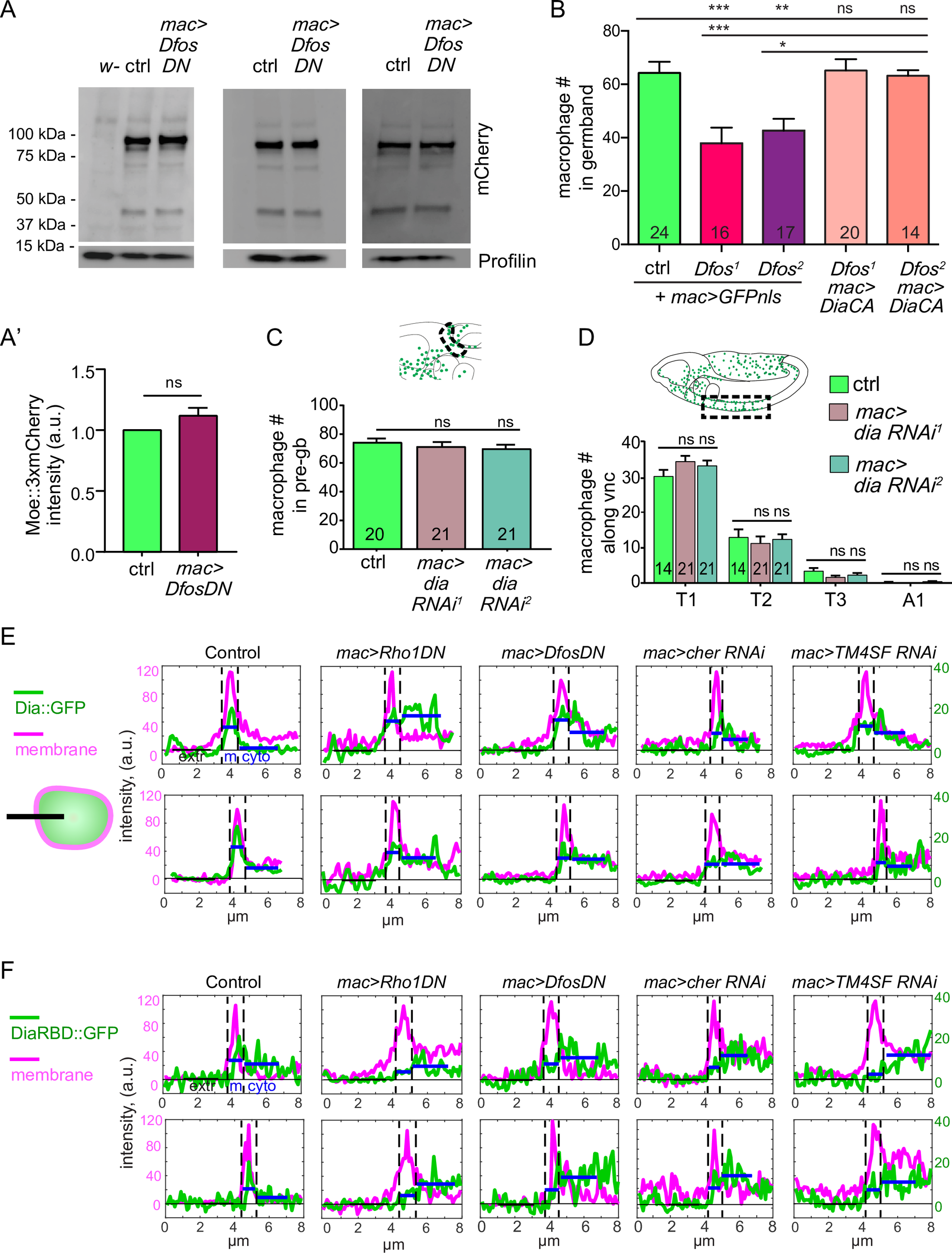

Supplement: S4 Fig — (A) Three western blots probed with an mCherry antibody of St 11 embryo extracts from srpHemo-moe::3xmCherry expressing either CD8::GFP (ctrl) or DfosDN in macrophages. Left western blot also contains w- lane. Original uncropped western blots can be found in S1 Raw images. (A’) Quantitation of the western blots. We observed no significant change in the expression of the Moe protein reporter when Dfos function is inhibited. (B) Expressing Dia-CA in macrophages in Dfos1 or Dfos2 embryos completely rescued the macrophage gb invasion defect. p-values: Control vs. Dfos1 or vs. Dfos2 p = 0.0004 or p = 0.0055, respectively; Control vs. Dfos1 mac>DiaCA or vs. Dfos2 mac>DiaCA p > 0.999; Dfos1 vs. Dfos1 mac>DiaCA p = 0.0005; Dfos2 vs. Dfos2 mac>DiaCA p = 0.035. SD: 20, 23, 18, 19, 7.8. (C, D) There was no significant change in the number of macrophages in (C) the pre-gb zone or (D) along the vnc in embryos expressing either of 2 different RNAi lines against dia expressed in macrophages. Pre-gb: Control vs. dia RNAi1 p = 0.54, Control vs. dia RNAi2 p = 0.77. vnc: Control vs. dia RNAi1 p = 0.99, Control vs. dia RNAi2 p = 0.95. RNAi1 = TRiP HMS05027, RNAi2 = TRiP HMS00308. (C) SD: 9, 12, 13. (D) SD: Ctrl 5.2, 6.4, 2.5, 0.4; dia RNAi1 5.6, 6.8, 1.7, 0.2; dia RNAi2 5.1, 4.9, 2.1, 0.6. (E, F) Two further examples of line profiles used for the determination of the membrane-to-cytoplasmic ratios in Fig 4N and 4P. Line intensity profiles of (E) Dia::GFP or (F) DiaRBD::GFP (green) and membrane myr::Tomato (magenta) across the edge of macrophages expressing either lacZ (Control), RhoDN, DfosDN, cher RNAi, or TM4SF RNAi as shown in the schematic in E. Line length approximately 8 μm. Blue lines indicate mean GFP intensity on the membrane and in cytoplasm. Histograms show mean ± SEM ***p < 0.005, **p < 0.01, *p <0.05. One-way ANOVA with Tukey post hoc was used for statistics of quantification. The number in each column corresponds to the number of analyzed embryos. “mac>” indicates srpHemo [file pbio.3001494.s009.tif]

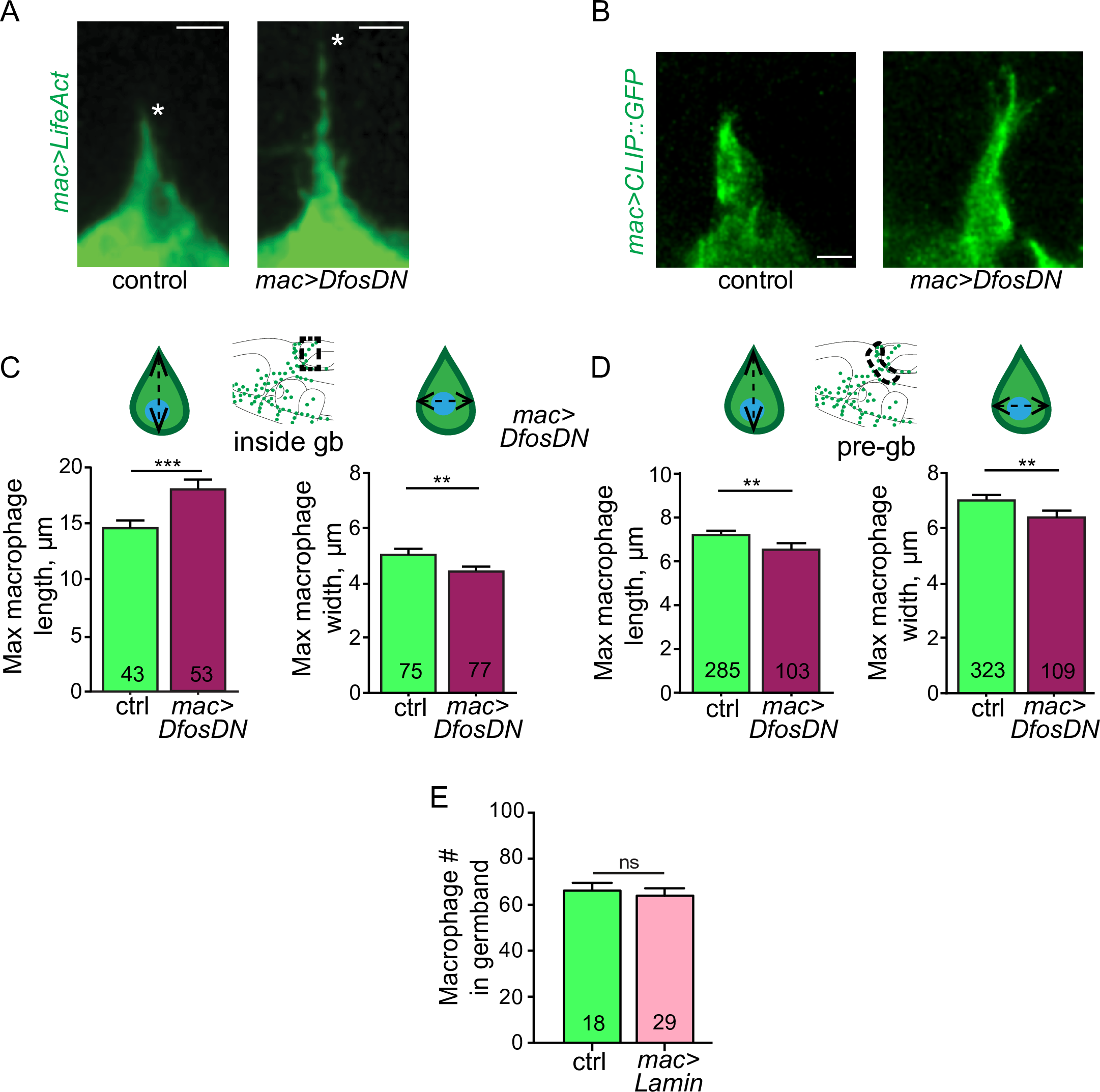

Supplement: S5 Fig — (A) Representative image showing actin protrusions of the first macrophage entering the gb in the control and in lines expressing DfosDN in macrophages. Actin was visualized by srpHemo-Gal4 (“mac>”) driving UAS-LifeActGFP. White stars indicate the tip of each actin protrusion. Scale bar 5 μm. (B) Microtubules are labeled with srpHemo-Gal4 driving UAS-CLIP::GFP. Spatially matched stills of the first macrophage expressing DfosDN and control extending protrusions into the gb slightly before entering with the body of the cell. As DfosDN macrophages have a delay in entry, the stills from the DfosDN movie are from a later developmental time point than the control. (C) Quantification of macrophage maximum length and maximum width shows that DfosDN expressing macrophages are 23% longer and 12% thinner than wild-type macrophages inside the gb (indicated in schematic above by dashed box). Control vs. DfosDN maximum length p = 0.0005, SD: 3.4, 5.7; control vs. DfosDN maximum width p = 0.0025, SD: 1.3, 1.0. (D) Quantification of the maximum length and maximum width of macrophages in the pre-gb zone (indicated in schematic by dashed box) shows that macrophages expressing DfosDN are 9% shorter and 9% thinner than wild-type macrophages. Control vs. DfosDN maximum length p = 0.0095, SD: 2.2, 2.0; control vs. DfosDN maximum width p = 0.005, SD: 2.3, 1.9. (E) Overexpression of UAS-Lam in macrophages through srpHemo-Gal4 (mac>) causes no change in their number in the gb compared to the control. p = 0.65, SD: 15, 18. Histograms show mean ± SEM ***p < 0.005, **p < 0.01, *p < 0.05. Unpaired t test was used for statistics of quantification. The number of measurements per genotype is shown in each columns. The data underlying the graphs can be found in S1 Data. ctrl, control; gb, germband; ns, not significant; SD, standard deviation; SEM, standard error of the mean. (TIF) [file pbio.3001494.s010.tif]

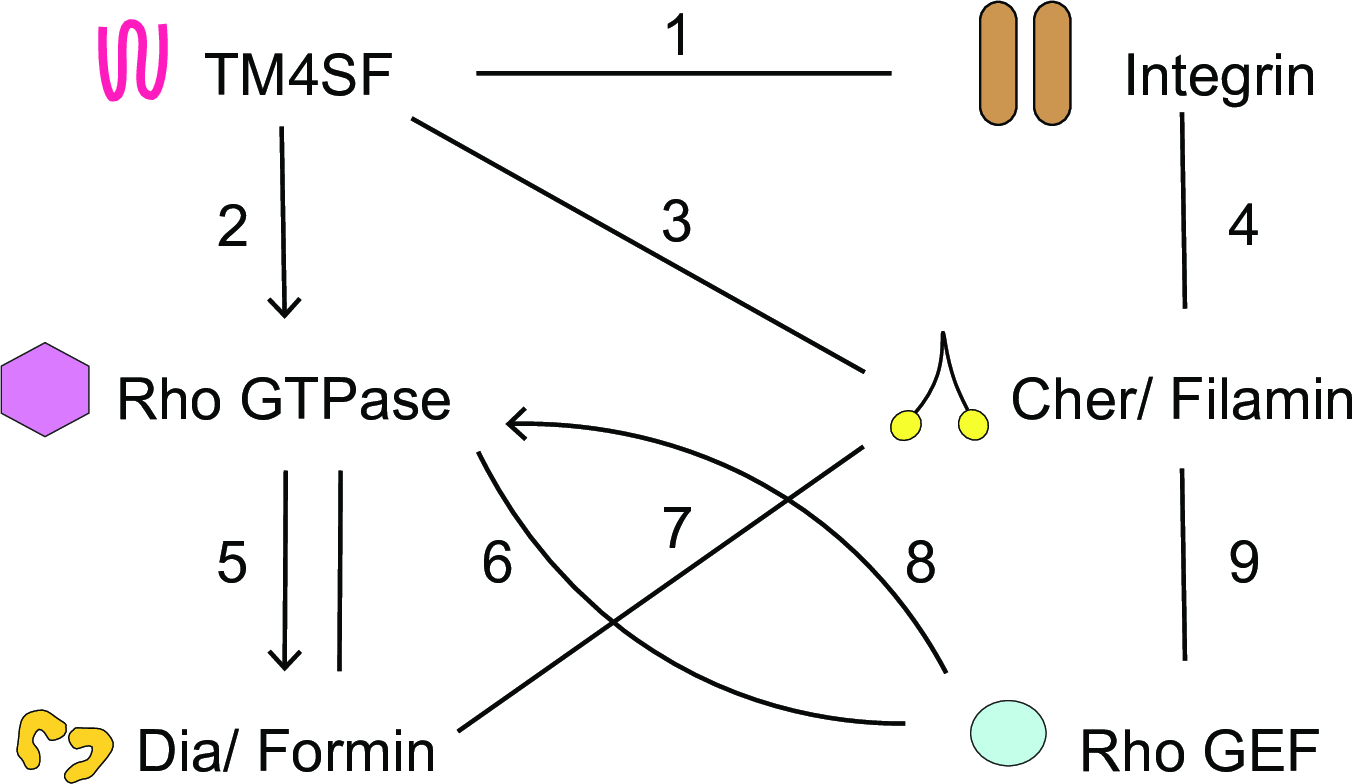

Supplement: S6 Fig — Proposed interactions of proteins at the cell cortex in wild-type macrophages during gb infiltration as shown in Fig 6. Direct binding between 2 proteins is indicated by a line, signaling between the interaction partners is represented as an arrow. These interactions and the resulting model in Fig 6 are based on the papers at the end of this legend next to the corresponding number shown for each linkage. The Tetraspanin TM4SF can cluster adhesion receptors such as Integrins at the membrane and lead to the recruitment and activation of Rho GTPases. Rho GTPases can bind and activate the formin Dia leading to F-actin polymerization. In addition, Integrin can bind filamins (Cher), which can bind to and thereby recruit RhoGEF to the membrane. Rho GEFs can in turn bind to and activate Rho GTPases. References for listed interactions: 1, Tetraspanins-Integrin) [71,76]. 2, Tetraspanins-Rho GTPases) [72–74]. 3, Tetraspanins-Filamins) [49,50]. 4, Integrin-Filamins) [35,68]. 5, Rho1 GTPase-Dia in Drosophila) [45,81]. 6 and 8, Rho GEF-Rho GTPases) [83]. 7, Formins-Filamins) [85,86]. 9, Filamins-RhoGEFs) [47,48]. Cher, Cheerio; gb, germband. (TIF) [file pbio.3001494.s011.tif]
