# Supplementary material for: Fos regulates macrophage infiltration against surrounding tissue resistance by a cortical actin-based mechanism in Drosophila
Source: PLoS Biol. 2022 Jan 6;20(1):e3001494. doi: 10.1371/journal.pbio.3001494 (PMC8735623; doi:10.1371/journal.pbio.3001494)
Supplement: S1 Raw images — Three original uncropped western blots of St 11 embryo extracts from srpHemo-moe::3xmCherry expressing either CD8::GFP (ctrl) or DfosDN in macrophages. Rightmost western blot also contains a w- lane. Top row shows blots probed with an mCherry antibody, bottom row the same blots probed with a profilin antibody as a loading control. Cropped versions of the blots are shown in S4A Fig. (PDF) [file pbio.3001494.s003.pdf]

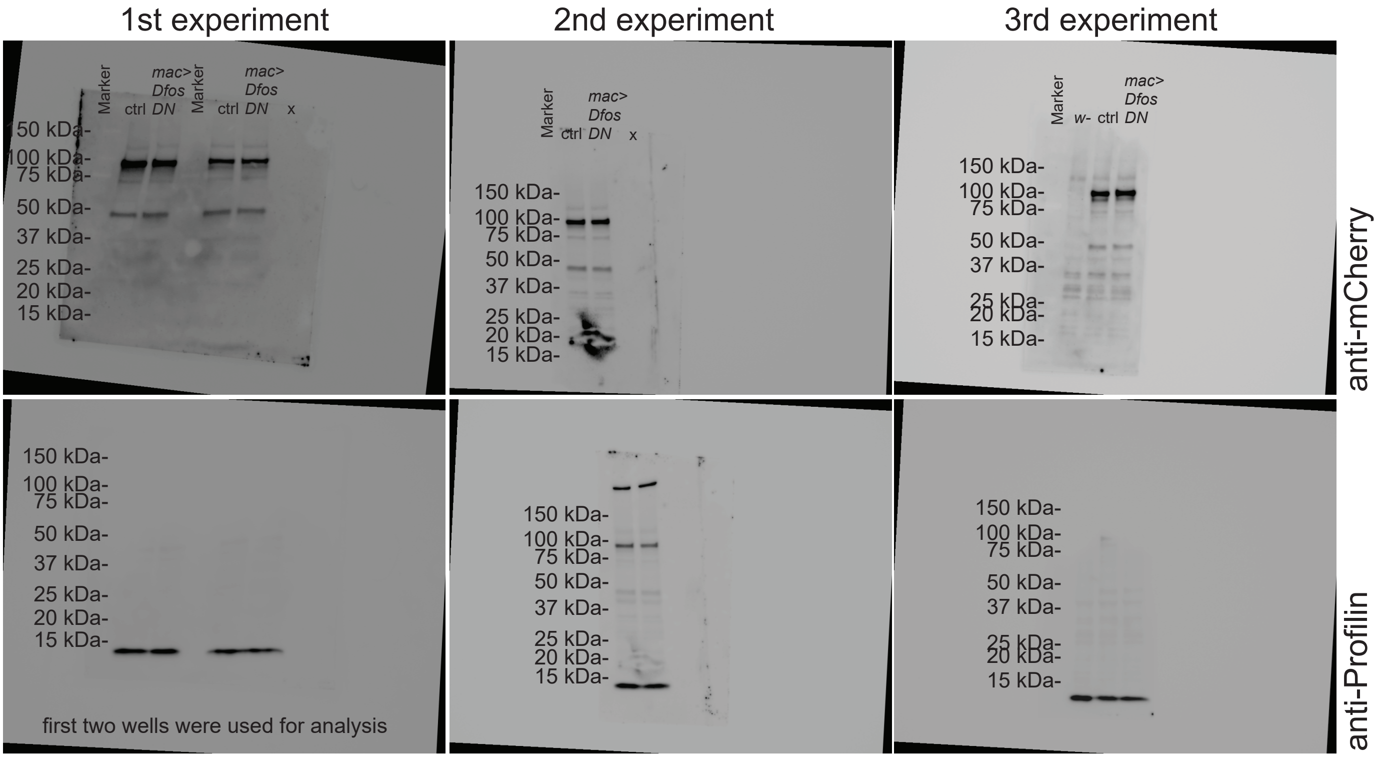

Chemilumienscence was recorded with a ChemieDoc MP (BioRad) molecular imager and the bands were densitometrically analyzed with ImageJ.

These blots are the source for the data shown in S4A-A'.
